# Discovery of a small molecule ligand of FRS2 that inhibits invasion and tumor growth

**DOI:** 10.1007/s13402-022-00753-x

**Published:** 2022-12-10

**Authors:** Karthiga Santhana Kumar, Cyrill Brunner, Matthias Schuster, Levi Luca Kopp, Alexandre Gries, Shen Yan, Simon Jurt, Kerstin Moehle, Dominique Bruns, Michael Grotzer, Oliver Zerbe, Gisbert Schneider, Martin Baumgartner

**Affiliations:** 1 Pediatric Molecular Neuro-Oncology Research Laboratory, Children’s Research Center, University Children’s Hospital Zürich, Zurich, Switzerland; 2 Present Address: Invasight, Zurich, Switzerland; 3 ETH Zurich, Department of Chemistry and Applied Biosciences, RETHINK, Zurich, Switzerland; 4 Department of Chemistry, University of Zurich, Zurich, Switzerland; 5 Department of Oncology, Children’s Research Center, University Children’s Hospital Zurich, Zurich, Switzerland; 6 ETH Singapore SEC Ltd, Singapore, Singapore

**Keywords:** FRS2, FGFR, Protein–protein interaction interference, Bioactive small molecule compound, Thermal proteome profiling, Cell invasion

## Abstract

**Purpose:**

Aberrant activation of the fibroblast growth factor receptor (FGFR) family of receptor tyrosine kinases drives oncogenic signaling through its proximal adaptor protein FRS2. Precise disruption of this disease-causing signal transmission in metastatic cancers could stall tumor growth and progression. The purpose of this study was to identify a small molecule ligand of FRS2 to interrupt oncogenic signal transmission from activated FGFRs.

**Methods:**

We used pharmacophore-based computational screening to identify potential small molecule ligands of the PTB domain of FRS2, which couples FRS2 to FGFRs. We confirmed PTB domain binding of molecules identified with biophysical binding assays and validated compound activity in cell-based functional assays in vitro and in an ovarian cancer model in vivo. We used thermal proteome profiling to identify potential off-targets of the lead compound.

**Results:**

We describe a small molecule ligand of the PTB domain of FRS2 that prevents FRS2 activation and interrupts FGFR signaling. This PTB-domain ligand displays on-target activity in cells and stalls FGFR-dependent matrix invasion in various cancer models. The small molecule ligand is detectable in the serum of mice at the effective concentration for prolonged time and reduces growth of the ovarian cancer model in vivo. Using thermal proteome profiling, we furthermore identified potential off-targets of the lead compound that will guide further compound refinement and drug development.

**Conclusions:**

Our results illustrate a phenotype-guided drug discovery strategy that identified a novel mechanism to repress FGFR-driven invasiveness and growth in human cancers. The here identified bioactive leads targeting FGF signaling and cell dissemination provide a novel structural basis for further development as a tumor agnostic strategy to repress FGFR- and FRS2-driven tumors.

**Supplementary Information:**

The online version contains supplementary material available at 10.1007/s13402-022-00753-x.

## Introduction

Small bioactive molecules precisely targeting receptor tyrosine kinases that promote tumor cell growth and invasion may lead to successful anti-metastatic therapies. Ideally, to overcome known resistance mechanisms, such lead structures should provide new activities to complement currently used drugs that target protein kinase catalytic domains. Successful leads may be molecules that specifically disrupt protein–protein interactions (PPI) involved in pro-invasive signal transmission downstream of the activated receptor tyrosine kinases. Small molecule PPI modulators can interact not only with protein–protein interaction interfaces (orthosteric inhibition) but also with allosteric sites (allosteric inhibition) [1], which will greatly increase their utility.

The diversity of potential allosteric interaction sites complicates the identification of effective and specific leads. An efficient method to explore the chemical space of the target and to select putative PPI ligands from compound libraries is virtual screening [2, 3]. Main challenges in developing effective PPI-inhibitory drugs are i) the identification of the disease-related and druggable PPIs, ii) the validation of the functional efficacy of the identified PPI inhibitor and iii) the prevention of potential off-target activities of PPI inhibitors. In this study, we addressed these challenges to identify a small molecule ligand of the adaptor protein fibroblast growth factor receptor substrate 2 (FRS2). FRS2 was recently identified to drive dissemination and tissue invasion in medulloblastoma (MB) [4], the most common malignant brain tumor in children [5].

Invasion-promoting activities of activated fibroblast growth factor receptors (FGFRs) require the binding of FRS2 to the juxtamembrane region of FGFRs [6–8], which couples FGFRs to downstream effectors and activates FGF signaling. We hypothesized that FRS2-FGFR interactions constitute a potentially druggable PPI with clinical relevance related to oncogenic signaling through FGFRs, where oncogenic alterations including amplification, mutation and fusion are common across human cancers [9–18]. FGFRs signal via FRS2-dependent (RAS/MAPK and PI3K/AKT) and FRS2-independent (PLC-γ, JAK-STAT) pathways [19]. FRS2 interacts with FGFRs via its phosphotyrosine-binding (PTB) domain [20] and increased expression or activation of FRS2 is involved in tumorigenesis of several tumor entities [21–25]. Selective (AZD4547, NVP-BGJ398 and JNJ-42756493) and non-selective (dovitinib or ponatinib) FGFR inhibitors have been explored for cancer therapy [26, 27]**.** However, resistance to FGFR inhibitors can evolve similarly to other receptor tyrosine kinase (RTK) inhibitors, either by the formation of gatekeeper mutations in the catalytic domain or the activation of bypass mechanisms [28, 29]. Targeting the adaptor protein FRS2 instead would likely prevent the evolution of FGFR gatekeeper mutations and could possibly also be effective in gatekeeper mutant FGFR-driven tumors by blocking signaling downstream of the RTK [26]. Moreover, specific targeting of FRS2 could reduce toxicities associated with FGFR inhibitor treatments, allow for a more precisely tuned repression of FGFR signaling, and may also target FGFR-independent oncogenic functions of FRS2. Repression of FRS2 function was previously explored via the inhibition of the FRS2-directed N-myristoyltransferase, which repressed FGFR signaling, cell proliferation and migration in several cancer types [30]. However, due to the more widespread activities of N-myristoyltransferases, their inhibition likely results in detrimental off-target activities.

In this study, we identified small molecule ligands of the PTB domain of FRS2 by selecting compounds that satisfy a pharmacophore model using computational (*‘*in silico*’*) screening methodologies. We functionally tested putative hits at the molecular, cellular, and organismal levels to identify novel lead structures that bind to FRS2 and interfere with FGF signaling. We furthermore validate targeted interference at the level of the RTK-adaptor protein interface as a novel strategy to block signal transmission from activated wild-type or fusion receptor tyrosine kinase.

## Results

In what follows we first describe the computational approach to design small molecules that bind to FRS2. We then used biophysical methods to characterize the interaction strength and describe basic features of the pharmacophore. NMR techniques are utilized to validate binding in a 1:1 ligand-receptor system and to locate the binding site. We subsequently describe cell-based assays to demonstrate that the leads do indeed suppress FGF-induced tumor invasion by interfering with downstream signaling. We describe pharmacokinetic properties of the molecules and show that the molecules suppress tumor growth in vivo and display little off-target activity.

### Discovery of small molecule compound ligands of the FRS2-PTB domain

**Fig. 1:**
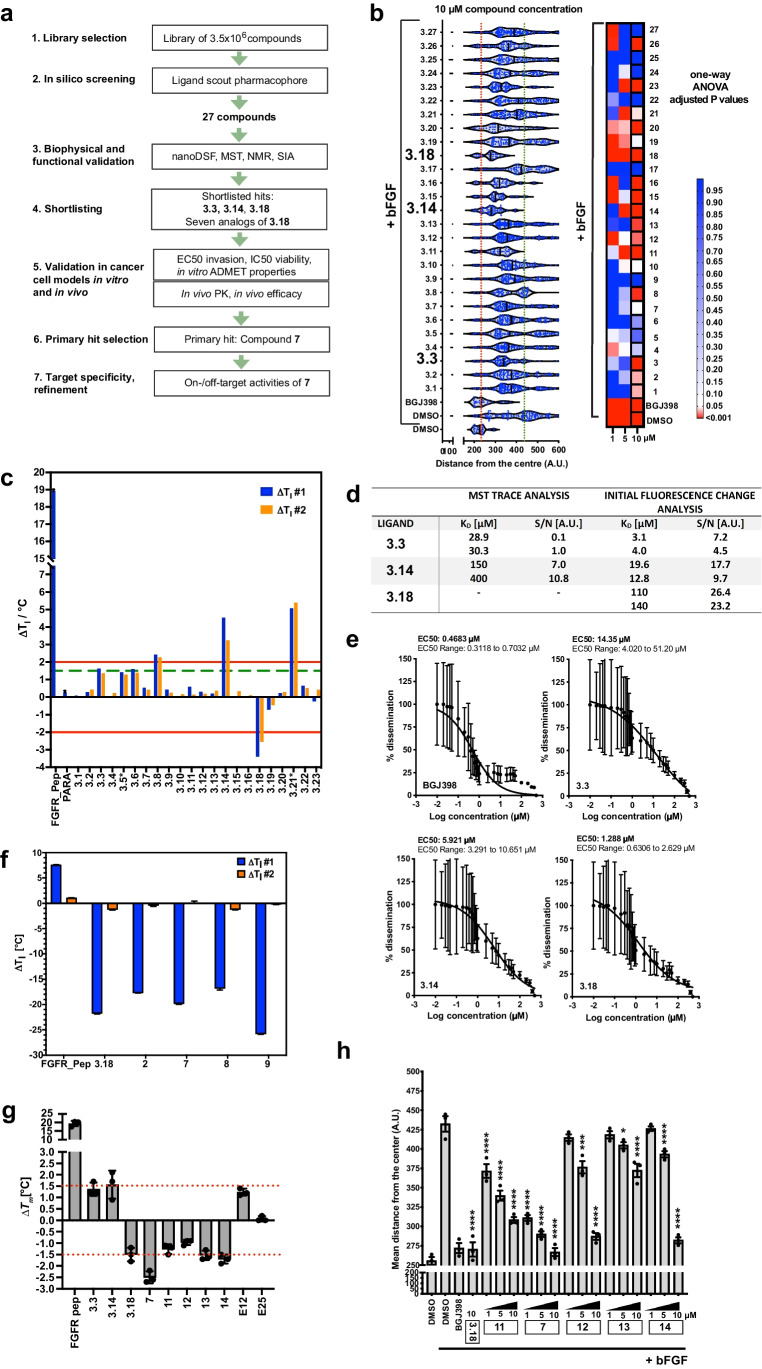
Identification and validation of small molecule compound ligands of the FRS2-PTB domain. **a**) Workflow of experimental procedures. **b**) Quantification of representative spheroid invasion assay (SIA) in DAOY cells treated with indicated compounds. Violin plot with median and quartiles of distances of invasion at 10 µM and adjusted *P* value from n = 3 technical replicas at 1, 5 and 10 µM compound concentrations are shown. Red dotted line: Maximal repression of bFGF-induced invasion, green dotted line: Maximal bFGF-induced invasion. **c**) nanoDSF analysis and compound-induced ΔT_m_ of FRS2_PTB of two independent measurements are shown. * marks compounds with self-fluorescence exceeding the protein’s fluorescence. Para: paracetamol. **d**) K_D_ of shortlisted compounds binding to GB1-FRS2_PTB determined by measuring change in initial fluorescence using MST trace analysis. Fitting with a signal–noise ratio < 5 is considered no binding. **e**) EC50 of shortlisted compounds for the inhibition of bFGF-induced collagen I invasion in DAOY cells. Log(Y) transformed and normalized invasion distances and corresponding SD of n = 3 technical replicas are plotted against compound concentrations. **f**) nanoDSF analysis of GB1-FRS2_PTB binding of **3.18** analogs. Mean and SD of compound-induced ΔT_m_ at 10 µM compound concentration is shown. n = 3 technical replicas. **g**) nanoDSF analysis of FRS2_PTB binding of shortlisted hits and bioisoesters of **3.18** and **7**. Mean and SD of compound-induced ΔT_m_ is shown of n = 3 technical replicas. **h)** Quantification of SIA in DAOY cells with bioisosteres of compounds **3.18** (**11**) and **7** (**12**, **13**, **14**) at 1, 5 and 10 µM compound concentrations. Mean and SD from n = 3 biological replicas are shown

The surface of the FRS2-PTB domain is relatively shallow and the interaction surface with the FGFR-peptide reportedly large [8]. A collection of 3.5 million compounds provided by multiple suppliers was virtual screened using LigandScout software [31] (Fig. 1a). The computational screening was performed against three-dimensional pharmacophore models derived from two pockets, one formed by the alpha-helix (A94-M105) and the beta-sheet (L33-L47), the other being complementary to the FGFR-peptide sequence I_pep_13-R_pep_16. The screening resulted in 14′217 hits (0.4% virtual hit rate), covering LigandScout pharmacophore scores of 52.0 – 76.0. A selection to maximize chemical diversity and to exclude excessive nitro and fluorine groups was performed that resulted in 27 compounds (Fig. S1a, Supplementary Table 1). We additionally virtually screened a collection of 7.68 × 10^4^ compounds assembled from different smaller libraries (“E series”) using the same pharmacophore models. The virtual screening of the E-series resulted in an approximately 0.2% virtual hit rate, and covered LigandScout pharmacophore-scores of 66.3—63.5. Nine of these E-series compounds were selected for further downstream analysis (Fig. S2a).

### Compound ligands are functionally active and bind to the FRS2-PTB domain

We tested the 27 selected (Fig. 1b) and 9 E series compounds (Fig. S2b) using the spheroid invasion assay (SIA) [32], which measures the capability of cells to invade a collagen matrix. Out of the 27 selected compounds, we shortlisted 22 and tested the predicted interaction between the compounds and the FRS2-PTB domain by nano differential scanning fluorimetry (nanoDSF) analysis. FRS2-PTB domains used for binding studies are depicted (Fig. S2c). The addition of the FGFR peptide (HSQMAVHKLAKSIPLRRQVTVS, Fig. S2c), which corresponds to the binding sequence of the natural ligand of FRS2, stabilized the PTB domain as it shifted the inflection point of the melting curve by + 19 ± 0.1 °C (Fig. 1c). We identified four compounds (**3.8**, **3.14**, **3.18** and **3.21**) that surpassed the threshold of a 2.0 °C shift, with one compound that led to a strong negative shift of greater than -2 °C (**3.18**, Fig. 1c). Compounds **3.14** and **3.18** were considered true hits. Compound **3.3** led to a modest shift of close to 1.5 °C and **3.8**, despite a clear positive temperature shift, displayed only moderate functional activity in the SIA (Fig. 1b). Compound **3.21** showed an auto-fluorescence (*) that exceeded the protein’s intrinsic fluorescence and was thus excluded. To increase the stability and solubility of the FRS2_PTB protein, we fused the highly soluble B1 immunoglobulin binding domain of the streptococcal protein G (GB1) to its N-terminus (Fig. S2c). Addition of the FGFR peptide to GB1-FRS2_PTB caused a shift in the melting temperature of + 10.5 ± 0.1 °C, which confirmed the binding of the natural ligand to the GB1-fused PTB domain (Fig. S2d).

To assess the affinity of interaction of the compounds with the FRS2-PTB domain, we performed microscale thermophoresis (MST) experiments using GB1-FRS2_PTB. We estimated the affinity of the FGFR peptide to the FRS2-PTB domain at 1.0 ± 0.2 μM by MST trace analysis. Compound **3.14** did show binding at signal:noise ratio > 5, whereas no binding was detected for **3.3** and **3.18** by MST trace analysis (Fig. S3a). In contrast, analysis of the fluorescence intensity change indicated clear binding of **3.14** and **3.18** to the PTB domain at signal:noise ratios > 5 for **3.14** and > 12 for **3.18**. **3.14** and **3.18** displayed affinities of 12.8–19.6 μM and of 110–140 μM, respectively (Fig. 1d). In conclusion, the results from the nanoDSF and MST analyses indicated that compounds **3.3**, **3.14** and **3.18** bind to the PTB domain of FRS2. Moreover, compounds **3.3**, **3.14** and **3.18** inhibited basic fibroblast growth factor (bFGF)-induced collagen I invasion of DAOY cells to a level similar to that caused by the FGFR inhibitor Infigratinib (BGJ398) [33]. A comparable inhibition of invasion was also observed for compounds **E12** and **E25** (Fig. S2b). Using bFGF-induced collagen I invasion as a measurand, we calculated the EC50 of BGJ398 (0.47 µM), **3.3** (14.35 µM), **3.14** (5.92 µM), **3.18** (1.29 µM), **E12** (18.15 µM) and **E25** (7.51 µM) (Figs. 1e, S2e) and focused our subsequent investigations on these compounds.

### Chloride and/or strong electronegative groups are necessary for optimal functional activity

Compound **3.18** displayed the most promising functional activity. To elucidate aspects of the structure–activity relationship of this hit, we tested ten commercially available structural analogs of **3.18** (1 –10) (Fig. S1b). Only seven of these compounds were soluble in aqueous solution and only four were soluble in 5% DMSO at the concentration required for the biophysical assays (**2**, **7**, **8** and **9**). A comparison of binding affinities to the FRS2-PTB domain using MST (Fig. S3a) and nanoDSF (Figs. 1f, S3b) revealed negative temperature shifts similar to **3.18** for all analogs (Fig. 1f). The binding of **3.18**, **2**, **7**, **8** and **9** was additionally tested in a nanoDSF competition assay with the FGFR peptide. We again measured a negative shift in compound-mixed samples, indicating that the peptide and compounds compete for FRS2-PTB binding (Fig. S3c). We then tested the functional activity of the seven soluble **3.18** analogs using SIA (Fig. S3d). We found that compound **7** performed equally well as **3.18**, whereas **8** displayed invasion inhibition only at the highest concentration. The remaining compounds caused less than 50% inhibition (Fig. S3d).

Both **7** and its parent compound **3.18** include a nitro group. To test the functional relevance of this group, we designed bioisosteres of **3.18** (**11**) and **7** (**12**, **13** and **14**), where the nitro group is replaced by either a carboxyl or a nitrile group (Fig. S1b). All tested bioisosteres displayed a negative temperature shift in the nanoDSF assay (Fig. 1g). At 10 µM concentration, compounds **12** and **14** reduced invasion comparable to the parent compound **7** (Fig. 1h). However, both compounds were less effective at 1 and 5 µM concentrations compared to **7**. **11** displayed a reduced effect compared to its parent compound **3.18** at all concentrations and **13** displayed a mild but dose-depended reduction of bFGF-induced collagen I invasion (Fig. 1h). This variable efficacy indicates that the position of the chloride group and/or of a strong electronegative group is important for activity.

### Selective inhibition of bFGF-induced invasion by shortlisted hits

Next, we evaluated the selectivity of the shortlisted hits by comparing compound activity on bFGF-induced cell invasion with compound activity on HGF- or EGF-induced invasion. All shortlisted compounds reduced bFGF-induced collagen I invasion by at least 50% (Fig. S4a). None of the compounds affected HGF-induced collagen I invasion (Fig. S4b). EGF-induced cell invasion was only moderately reduced by compounds **3.18**, **7**, **9** and **14** (Fig. S4c), indicating that the shortlisted hits effectively and predominately inhibit bFGF-induced collagen I invasion. Furthermore, the invasion of the non-bFGF dependent SHH MB tumor cell line ONS-76 remained unaffected by the treatments (Fig. S4d), together demonstrating selective inhibition of bFGF-induced cancer cell invasion (Fig. S4e). Combining the biophysical and functional analyses, we shortlisted the following hits as potential inhibitors of FRS2: **3.3**, **3.14**, **3.18**, **E12**, **7**, **13**, and **14**.

### Confirmation of compound-target binding by NMR

**Fig. 2:**
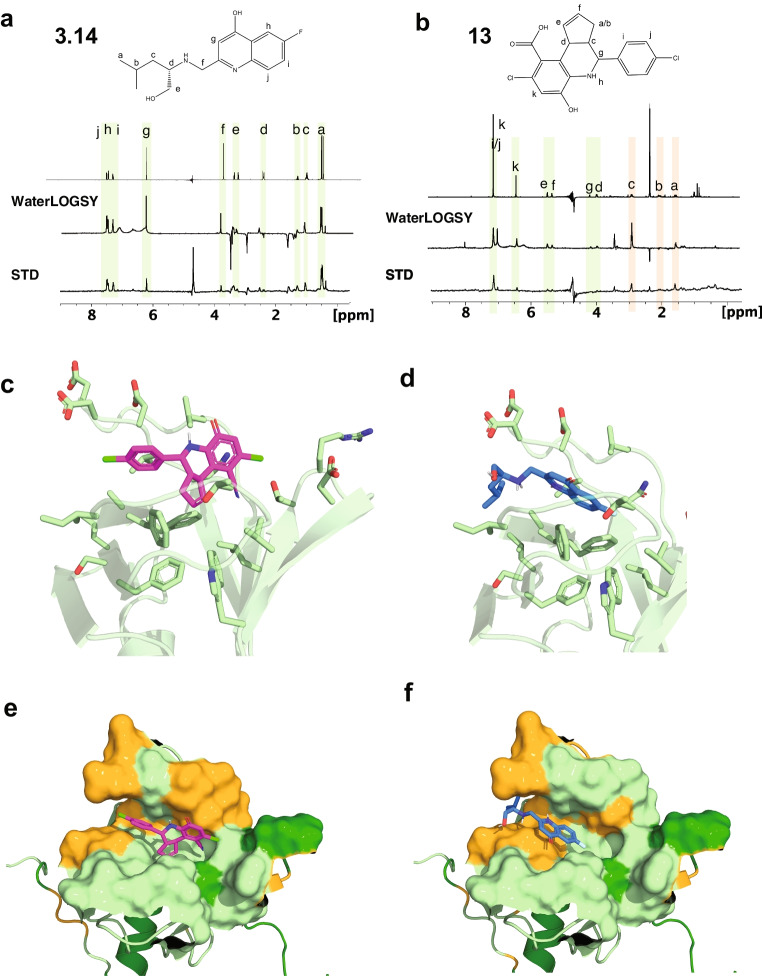
NMR screening of compounds **3.14** and **13**. Reference spectrum with assignments of **3.14 a**) and **13 b**) (upper trace) and WaterLOGSY (middle) and STD (bottom) of 25 µM GB1-FRS2-PTB and 500 µM **3.14** or **13** at 280 K. Resonances exhibiting protein-interaction in the STD and/or the WaterLOGSY spectra are highlighted in light green, while resonances that might be obscured by buffer signals are indicated by light orange strips. (**c** and **d**) Cartoon of secondary structure showing the backbone trace of FRS2_PTB with side-chains of the binding pocket depicted as sticks. Compounds **13** (**c** and **e**) and **3.14** (**d** and **f**) are depicted as purple and blue sticks. (**d** and **f**) Protein surface within a 8 Å sphere around the ligand atoms. Residues with significant CSPs are highlighted in orange. Unassigned residues are shown in dark green. Note that, additional WaterLOGSY signals with a negative signal phase and originating from the arginine of the buffer system were detected for **3.14**. These arginine resonances also interfered with the resonances a,b and c of compound **13**, rendering the corresponding WaterLOGSY and STD signals ambiguous

To confirm binding of shortlisted hits to the PTB domain of FRS2 in vitro*,* we performed solution nuclear magnetic resonance (NMR) analyses. First we used saturation transfer difference (STD) and water-ligand observed via gradient spectroscopy (WaterLOGSY) ligand observation experiments [34] to verify binding of the candidate molecules. Both the STD and the WaterLOGSY clearly demonstrated binding of the most soluble ligand **3.14** and of the bioisostere of **7**, compound **13**, to the PTB domain (Fig. 2a and b). The difference spectrum of the experiment displayed distinct STD signals for most of the proton resonances of compounds **3.14** and **13**, with most prominent signals due to the aromatic positions i,j and h in **3.14** or i and j in **13**. In addition, the WaterLOGSY spectra exhibited strong positive signals at the corresponding positions, verifying the compound-protein interaction (Figs. 2a and b, S5a and b).

To identify the binding site for the ligands in the FRS2-PTB domain, we used chemical shift perturbation (CSP) techniques derived from [^15^ N,^1^H-HSQC] spectra (Fig. S5c). We failed to reproduce refolding of FRS2 under various conditions (Fig. S5c_I_) using a construct encompassing only the PTB domain of FRS2 (FRS2_PTB; Fig. S2c) for which the structure was published previously [8]. While addition of the FGFR peptide, fusion to the small protein tag GB1 and the non-denaturing detergent CHAPS improved quality of the spectra (Fig. S5c_II_), signals from the GB1-FRS2_PTB construct remained weak (Fig. S5c_III_). To further stabilize peptide binding, we covalently fused the FGFR1 peptide either to the N- or the C-terminus of the PTB through a GGS linker. The C-terminal fusion of the FGFR peptide with GB1-FRS2_PTB (Fig. S2c) finally allowed to generate good-quality [^15^ N,^1^H]-HSQC spectra (Fig. S5c_IV_). With triple-resonance NMR in combination with ^2^H,^13^C,^15^ N protein labeling, we could assign almost all backbone resonances from residues of the β-sheets, except for the 6^th^ β-strand (Fig. S5d). Signals from the α-helix in general were either absent or so weak that we failed to connect them to neighboring residues, possibly because the helix is not sufficiently tightly packed against the β-sheet triggering conformational exchange effects.

Upon compound addition, we detected very small CSPs in the FGFR peptide fusion, likely because the putative bindings site is occluded by the C-terminally fused peptide. We therefore measured compound-induced CSPs on the non-covalent GB1-FRS2_PTB:FGFR peptide complex, for which the affinity of the FGFR peptide to the protein was lower. Most of the significant CSPs were in the loop connecting β-strands 1 and 2 that covers the cleft between the two halves of the β-sandwich, and in β-strand 3 (Fig. S5d and e). In general, CSPs are larger for compound **13**, which we attributed to the better aqueous solubility of the ligand. The CSPs are located remote to the targeted binding pocket and rather spread around an area that is occluded by the binding of the FGRF peptide. Since the FGFR peptide and the compounds share a common binding site, the observed CSPs are rather small. Consistently, the results obtained in the ligand-observe STD and WaterLOGSY experiments using constructs without the FGFR peptide were much clearer.

To investigate whether the ligands could potentially displace the peptide in this area, we removed the peptide coordinates and attempted docking of the ligands to the PTB domain, while allowing for small adjustments of the protein backbone and larger changes for its sidechains within the putative binding pocket. Predicted docking poses that are in agreement with the measured CSPs are shown for **3.14** and **14** (Fig. 2c-f). In all these poses, the aromatic rings of the ligands form π–stacking interactions with the two aromatic sidechains of Phe-87 and Phe-74. In addition, we observed several hydrogen bonds.

### Shortlisted hits inhibit FGFR-driven invasiveness of human cancer cell lines

**Fig. 3:**
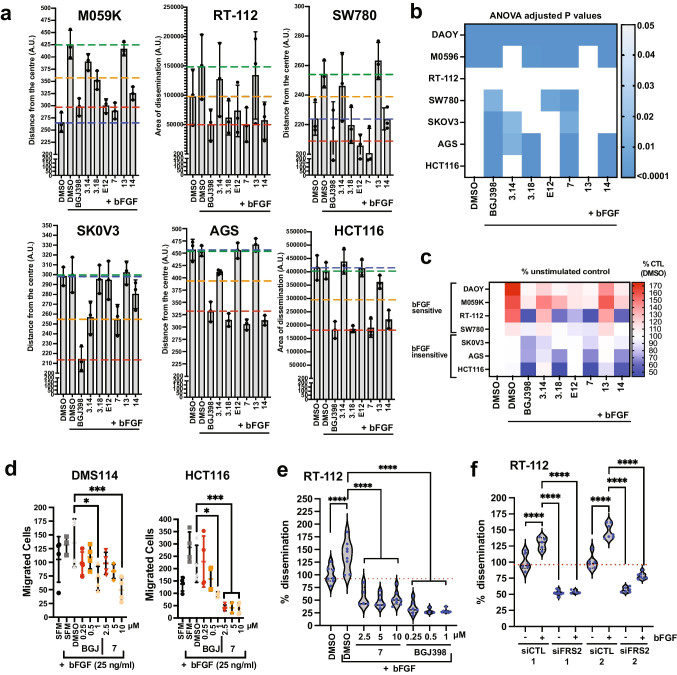
Compound-mediated blockade of collagen I invasion in human cancer cell models. **a**) SIA analysis with shortlisted compounds in different human cancer cell models. Mean distance or area (RT-112) of invasion and SD of *n* = 3 experiments at 10 µM compound concentrations are shown. Dotted lines: Blue: Basal mean invasion unstimulated, green: maximal mean invasion bFGF-stimulated, red: Maximal inhibition of invasion by BGJ398, orange: 50% of maximal bFGF-induced mean invasion. **b)** Heat map of adjusted *P* values of SIA analysis shown in a and in S4a for DAOY cells. **c**) Heat map of percent change of invasion relative to unstimulated control of SIA shown in a and in S4a for DAOY cells. **d**) Boyden chamber transwell migration assay. Mean and SD of n = 4 technical replicas and one-way ANOVA adjusted *P* value statistics are shown (* p =  < 0.05, *** p =  < 0.001). **e**) SIA of RT-112 cells comparing inhibitory effect of compound with BGJ398. **f**) SIA of RT-112 cells transfected with either two different FRS2-specific or their corresponding control siRNAs. Violin plot with median and quartiles of distances of invasion area of invasion of ≥ 6 spheroids and one-way ANOVA adjusted *P* value statistics from representative experiment are shown in e) and f) (**** p =  < 0.0001)

bFGF is a strong promoter of collagen I invasion in the established SHH MB cell line DAOY [4, 32]. Hence, we used this cell model for compound screening and shortlisting the hits. However, FGFR aberrations are common across human cancers and specific targeting of aberrant FGFR signaling is an established strategy against different types of cancer [26, 27]. Therefore, we tested the efficacy of compounds in inhibiting tumor-promoting functions in four FGFR-driven cancer cell lines: DMS114 (small cell lung cancer), HCT116 (colorectal carcinoma), SNU16 (gastric carcinoma), AGS (gastric adenocarcinomas). In addition, based on the FGFR3 fusions and overexpression of FRS2, we also included SW780 (urinary bladder carcinoma), RT-112 (urinary bladder carcinoma), M059K (glioblastoma) and SKOV3 (ovarian adenocarcinoma) cell lines. We confirmed the mRNA and protein expression levels of FRS2 and FGFRs in these cell lines using qPCR (Fig. S6a and b) and immunoblotting (IB, Fig. S6c), respectively. Next, we tested whether bFGF induced collagen I invasion of these cell lines using SIA. SNU16 and DMS114 cells did not invade the collagen I matrix upon stimulation with bFGF. The remaining cell lines were categorized as bFGF-sensitive (M059K, RT-112, SW780) or bFGF-insensitive (SKOV3, AGS and HCT116), depending on whether collagen I invasion was increased by bFGF stimulation (Fig. 3a and b). We then tested the effect of the shortlisted hits in these cell lines using the SIA. Compounds **7** and **14** inhibited collagen I invasion in both bFGF-sensitive and bFGF-insensitive cell lines (Figs. 3a-c, S6d). In contrast, **E12** inhibited collagen I invasion only in bFGF-sensitive cell lines M059K, RT-112 and SW780. Compound **3.14** caused approximately 50% inhibition in the SKOV3 and AGS cells and **3.3** displayed significant repression of collagen I invasion only in SKOV3 and M059K cells (Figs. 3a and b, S6d). DMS114 did not invade collagen I hydrogels. Therefore, we quantified basal and bFGF-induced migration of DMS114 with the Boyden transwell migration assay. We used the HCT116 cell line as control (Fig. 3d). bFGF caused only a minor increase in transwell migration of DMS114. However, treatment with compound **7** or BGJ398 caused a dose-dependent reduction of migration with a significant reduction observed at 10 µM and 1 µM, respectively. In HCT116 cells, bFGF stimulation caused a twofold increase in transwell migration, and both basal and bFGF-increased transwell migration was abolished with 2.5, 5 or 10 µM of compound **7** (Fig. 3d).

The bladder carcinoma cell line RT-112 expresses the FGFR3-TACC3 fusion, which results in ligand-independent activation of the FGFR [35]. We found that bFGF stimulation of these cells further increased collagen I invasion, and that treatment of these cells with compound **7** reduced both basal and ligand-induced collagen I invasion (Fig. 3e). To confirm that FRS2 is necessary for the transmission of basal and bFGF-induced invasion signals, we depleted FRS2 in RT116 cells with two different siRNAs. Consistent with a relevant role of FRS2 in these cells, we found that the siRNAs effectively repressed collagen I invasion, both under basal conditions and in the presence of bFGF (Fig. 3f). This not only confirms the relevance of FRS2 for the transmission of pro-invasive signaling from the fusion FGF receptor but also highlights the efficacy of **7** to interfere with pro-invasive FGFR3-TACC3 signaling in RT-112 cells.

In conclusion, these cell-based assays confirmed that compound **7** is functionally active in various cancer cell models and represses FGFR induced invasiveness. Importantly, compound **7** may also be effective in the inhibition of cancer cell invasiveness driven by FGFR3-TACC3 fusions.

### Compound 7 effectively inhibits FGFR-driven MAPK activation

We next tested the effect of the shortlisted hits on the viability of the cell lines grown either in 2D culture (Fig. S7a) or as 3D spheroids (Fig. S7b) using the CellTiter-Glo assay. We found that compounds **7** and **14** at a concentration between 2.7 and 20 µM reduced viability by more than 50% in DAOY, AGS and HCT116 cells after 48 h of treatment. BGJ398 reduced viability of SW780, RT-112, M059K and SKOV3 cells in this concentration range.

**Fig. 4:**
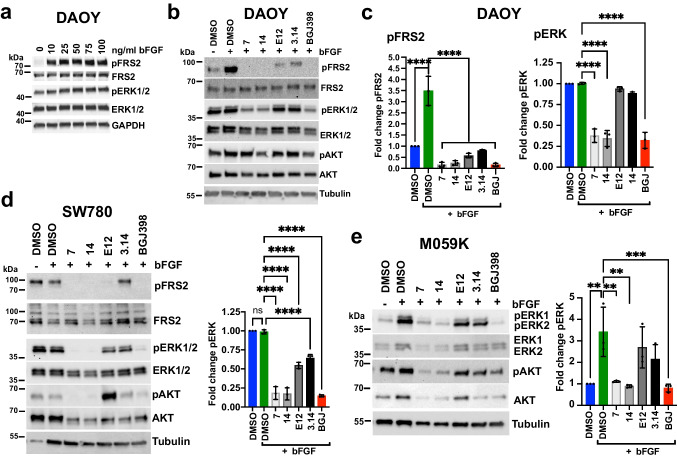
Compound-mediated blockade of MAPK pathway activation in human cancer cell models. **a**) IB analysis of bFGF-induced FRS2_Y436_ phosphorylation in DAOY cells. **b**) IB analysis of FRS2_Y436_, ERK1/2_Thr202/Tyr204_ and AKT_S476_ phosphorylation in compound treated DAOY cells stimulated with bFGF. **c**) Bar diagrams depicting the quantification of the phosphorylation of FRS2_Y436_ and ERK1/2_Thr202/Tyr204_ relative to unstimulated control. **d)** Left: IB analysis of ERK1/2_Thr202/Tyr204_ phosphorylation in compound treated SW780 cells stimulated with bFGF. Right: Bar diagram depicting the quantification of ERK1/2_Thr202/Tyr204_ phosphorylation relative to unstimulated control. **e**) Left: IB analysis of ERK1/2_Thr202/Tyr204_ and AKT_S476_ phosphorylation in compound treated M059K cells stimulated with bFGF. Right: Bar diagram depicting the quantification of ERK1/2_Thr202/Tyr204_ phosphorylation relative to unstimulated controls. Bar diagrams in c-e show mean fold change of phosphorylation of n = 3 independent experiments, SD and one-way ANOVA adjusted *P* values of comparison to SFM + bFGF are shown

Next, we evaluated short-term treatment effects of the shortlisted hits on the downstream effectors of bFGF signaling using immunoblotting. bFGF stimulation increases phosphorylation of FRS2 (Y436) in DAOY cells [4] (Fig. 4a). All compounds reduced basal or bFGF-induced Y436 phosphorylation of FRS2 in DAOY or SW780 cells, except **3.14**, which was not effective in SW780 cells (Fig. 4b-d). Compounds **7**, **14** and BGJ398 inhibited basal and bFGF-induced phosphorylation of extracellular regulated kinase ERK (pT202/Y204) in DAOY, SW780, M059K, SKOV3, AGS, DMS114 and RT-112 cells (Fig. 4b-e, S7c-g). **3.14** significantly reduced pERK in SW780 and AGS cells (Figs. 4d, S7d). In RT-112 cells, compounds **7** and **14** and BGJ398 reduced basal and bFGF-induced phosphorylation of FRS2_Y436_ (Fig. S7g). Compound effects on phosphorylation of AKT(pS473) were inconclusive and in **E12**-treated SW780 cells, we even observed an increase in pAKT (Fig. 4d). These data demonstrate functional activity of shortlisted hits in repressing growth and FGFR signaling across a panel of cancer cell lines and confirm the superior inhibitory efficacy of compound **7** and its bioisostere **14**.

### Pharmacokinetic properties of compounds E12, 3.14 and 7 and maximum tolerated dose of compound 7

**Fig. 5:**
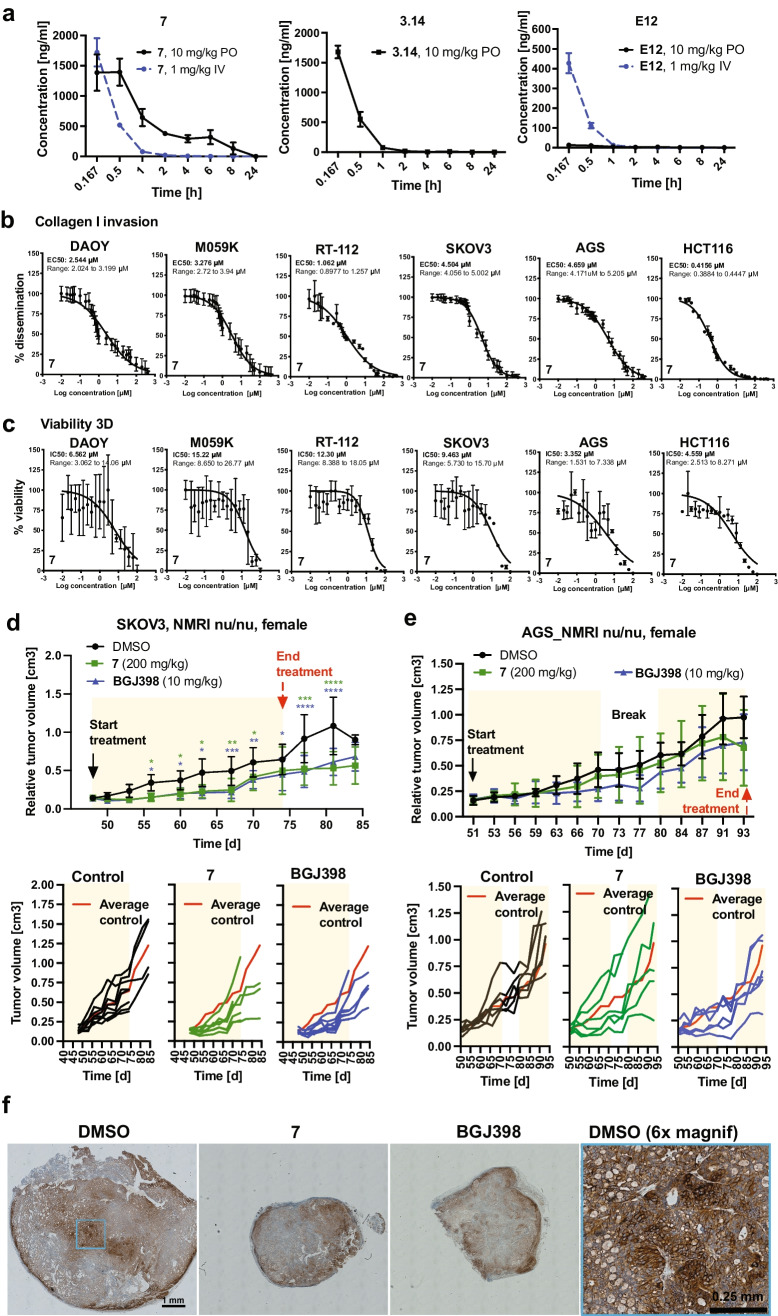
Bioavailability of compounds and in vivo efficacy of compound **7**. **a**) Plasma exposure levels after 10 mg/kg peroral (PO, compounds **E12** and **7**) or 1 mg/kg intravenous (IV) (compounds **E12**, **3.14** and **7**) administration in mice quantified at intervals between 0.167 and 24 h. Means and SD of n = 3 animals are shown. **b**) EC50 curves of SIA with compound **7** in different human cancer cell models in the presence of 100 ng/ml bFGF. **c**) IC50 curves of CellTiter Glo cell viability analysis with compound **7** in different human cancer cell models in full growth medium. Log(Y) transformed and normalized invasion distances (b) or viability (c) and corresponding SD of n = 3 technical replicas are plotted against compound concentrations. **d**) Tumor volumes of SKOV3 flank tumors of mice treated with DMSO, 10 mg/kg BGJ398 or 200 mg/kg compound **7** relative to volumes at start of treatment. A two-way ANOVA analysis with Turkey’s multiple comparison test was performed (* p < 0.05, **p < 0.01, ***p < 0.001, ****p < 0.0001). Lower: Tumor growth curves of the individual tumors. **e**) Upper: Tumor volumes of AGS flank tumors of mice treated with DMSO, 10 mg/kg BGJ398 or 200 mg/kg compound **7** relative to volumes at start of treatment. Lower: Tumor growth curves of individual tumors. **f**) IHC analysis of pFRS2_Y436_ staining in tumor samples of the SKOV3 flank tumor model at d74 (end of treatment)

We determined in vitro pharmacokinetic (PK) properties for compounds **E12**, **3.14** and **7** (Supplementary Table 2). **E12** and **3.14** are freely soluble and show a t_1/2_ of clearance (CL_1/2_) of 5.52 and 29 min, respectively. **E12** displayed moderate toxicity and **3.14** no toxicity in the tetrazolium salt reduction (MTT) assay. Compound **7** is sparingly soluble, shows a CL_1/2_ of 3.62 min and is moderately toxic in the MTT assay. We nevertheless decided to test in vivo PK for these three compound classes. We determined exposure levels by quantifying compound concentrations in plasma samples at various intervals after per oral (PO) or intravenous (IV) administration (Fig. 5a, Supplementary Table 3). **E12** showed a CL_1/2_ of 2.71 h but only low oral bioavailability of 13 ng/ml and an area under the plasma concentration–time curve from time zero to time of last measurable concentration (AUClast) of 22 h*ng/ml. **7** showed oral bioavailability with a C_max_ of 1395 ng/ml, a CL_1/2_ of 3.83 h and an AUClast of 3333 h*ng/ml. After IV administration, C_0_ of **E12** was relatively low (762 ng/ml), CL_1/2_ was only 0.15 h and AUClast was 85 h*ng/ml. Both **3.14** and **7** displayed considerably better bioavailability (C_0_ of 2703 and 2873 ng/ml, respectively), a CL_1/2_ of 1.55 and 1.14 and an AUClast of 413 and 398 h*ng/ml, respectively (Supplementary Table 3). The relatively high oral bioavailability, satisfying CL_1/2_ and AUClast after oral gavage of **7** prompted us to focus on this compound for further studies.

To establish the effective concentration for in vivo dose finding, we compared the EC50 of compound **7** in the SIA invasion analysis (Fig. 5b) with the IC50 of **7** in 2D (Fig. S8a) and 3D (Fig. 5c) cell viability assessed by the CellTiterGlo assay. We used three bFGF-sensitive (DAOY, M059K and RT116) and three bFGF-insensitive (SKOV3, AGS and HCT116) cell models. For all but AGS was the EC50 of the SIA lower than the IC50 for cell viability. This indicated a selective sensitivity of most cell models tested to compound **7**-mediated inhibition of cell invasiveness, and that the reduction in collagen invasion observed in compound **7**-treated cells was most likely not due to toxicity. The IC50 of compound **7** was for all lines below 20 µM, suggesting that this concentration should both block invasion and reduce viability across all models.

We next determined the in vivo single-dose and multi-dose maximum tolerated dose (MTD) and in vivo PK of compound **7**. Animals received an initial dose of 30, 100 or 200 mg/kg PO. No mortality and body weight loss was observed, signifying that the tested doses were tolerated after the single administration (Fig. S8b). For the multi-dose MTD, compound **7** was administered daily for 5 days, at 200 mg/kg. This treatment induced vocalization, hunchback and decrease in touch response, abdominal and limb tone and low limb post throughout the experimental period. No mortality was noted, but a 10% body weight loss was observed on days 1—5 and an additional 14.6% was found from days 5 – 8. Plasma and liver samples from mice treated with compound **7** were harvested at 0.5 h after administration and the levels of **7** were determined by LC–MS/MS to determine exposure levels. With the 200 mg/kg administration, the desired plasma concentration of 10 µg/ml (> 20 µM) was reached. Mice were then treated once daily for 5 days and compound concentrations in plasma samples were determined at day five 0.5, 1, 2 and 6 h after compound administration (Fig. S8c). This multi-dose study demonstrated that a plasma concentration of 7.5 mg/ml (20 µM) was reached for at least six h after administration, and we decided to use this concentration for the in vivo study.

### Compound 7 blocks cancer growth in vivo

We used SKOV3 and AGS mouse xenograft models to assess in vivo efficacy of compound **7**. Tumor cells were flank injected and BGJ398 was used as positive control. Animals bearing palpable tumors were randomized in three groups, and then treated once per day PO with either vehicle (group A), 200 mg/kg compound **7** (group B) or 10 mg/kg BGJ398 (group C) (Fig. S8d and g). Treatment of AGS tumor-bearing mice was interrupted for 7 days at day 72 post implantation due to signs of toxicity in the compound **7**-treated animals (Fig. S8g). Tumor volumes (TV) and body weight (BW) was measured before, throughout and after treatment. Compound **7** treatment caused a significant reduction in tumor growth between d55 and d80 in the SKOV3 model (Fig. 5d upper, S8e and f). Reduction was comparable to BGJ398 treatment effect, and six out of the seven compound **7**-treated mice displayed less than average control tumor growth during treatment (Fig. 5d, lower). However, some increased tumor growth was observed in compound **7**-treated animals after d67 (Fig. 5d). In the AGS model, compound **7** treatment did not significantly reduce average TV compared to control (Fig. 5e, upper). Inspection of the individual tumor growth curves revealed that TV in four out of the six compound **7**-treated animals was reduced at d67 and plateaued between d70 and 77 (Fig. 5e, lower, S8h). No response was observed in two out of six animals. We also assessed FRS2_Y436_ phosphorylation by IHC analysis at the end of the treatment period (Fig. 5f). Tumors of control animals displayed a heterogenous pFRS2_Y436_ signal with signal enrichment in the periphery of the tumors and in central regions. No clear difference in pFRS2 _Y436_ was observed in tumor tissues of compound **7** or BGJ398-treated animals (Fig. 5f).

In conclusion, these data revealed a transient anti-tumor efficacy of compound **7** in the SKOV03 model in vivo. Efficacy of **7** is comparable to that of the structurally and functionally unrelated FGFR inhibitor BGJ398. We furthermore confirmed that pFRS2 can be detected in the tumor tissue by IHC analysis.

### Compound 7 acts on FRS2 and displays limited off-target activities

**Fig. 6:**
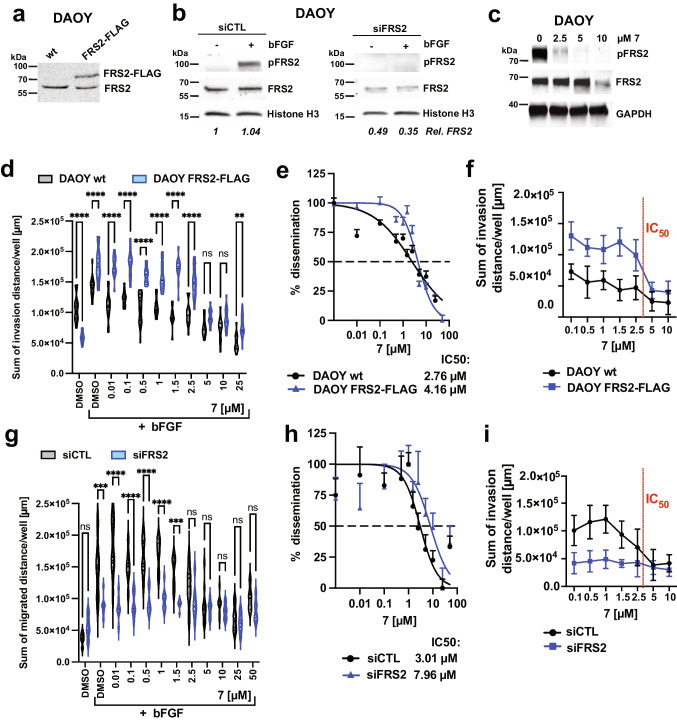
Level of FRS2 determines compound **7** efficacy. **a)** IB analysis of the expression levels of total endogenous FRS2 in DAOY wt cells in comparison to FRS2 expression in DAOY FRS2-FLAG cells. **b)** IB analysis of the expression and FRS_Y436_ phosphorylation levels of total endogenous FRS2 in DAOY siCTL cells in comparison to DAOY siFRS2 cells 48 h post transfection. Cells were either maintained in SFM or in SFM supplemented with 100 ng/ml bFGF for five min. **c)** Validation by IB analysis of compound **7** activity towards bFGF-induced FRS2_Y436_ phosphorylation in DAOY cells. **d)** Comparison of compound **7** repression of bFGF-induced collagen I invasion in DAOY wt and DAOY FRS2-FLAG cells by SIA. Truncated violin plot with median and quartiles of distances of invasion at increasing compound **7** concentrations is shown. A 2-way ANOVA test with Šídák's multiple comparison was performed (** p < 0.01 and **** p < 0.0001). **e)** Comparison of EC50 of collagen I invasion for compound **7** in DAOY wt and DAOY FRS2-FLAG cells. Log(Y) transformed and normalized invasion distances and corresponding SEM of n = 10 technical replicas plotted against compound concentrations are shown. **f)** Dose effect on cumulated invasion distances in DAOY wt and DAOY FRS2-FLAG cells. **g)** Comparison of compound **7** repression of bFGF-induced collagen I invasion in DAOY siCTL and DAOY siFRS2 cells. Violin plot with median and quartiles of distances of invasion at increasing compound **7** concentrations is shown. A 2-way ANOVA test with Šídák's multiple comparison was performed (** p < 0.01, *** p < 0.001 and **** p < 0.0001). **h)** Comparison of EC50 of collagen I invasion for compound **7** in DAOY siCTL and DAOY siFRS2 cells. Log(Y) transformed and normalized invasion distances and corresponding SEM of n = 10 technical replicas are plotted against compound concentrations. **i)** Dose effect on cumulated invasion distances in DAOY siCTL and DAOY siFRS2 cells. Means and SD of > 5 technical replicas are shown

We next explored whether efficacy of compound **7** is related to the expression levels of FRS2. Towards that, we performed a dose response analysis with **7** in DAOY cells that either co-express FRS2-FLAG (FRS2-FLAG), resulting in increased FRS2 expression (Fig. 6a), or in DAOY cells where we reduced FRS2 expression by siRNA, which resulted in complete repression of bFGF-induced FRS2_Y436_ phosphorylation (Fig. 6b). As quality control, we also confirmed the efficacy of the compound **7** batch used for these experiments (Fig. 6c). We found that DAOY cells co-expressing FRS2-FLAG invaded the collagen I matrix more efficiently compared to wt cells (Fig. 6d). Increasing the FRS2 expression levels also led to a moderate increase in the IC50 from 2.76 µM in wt to 4.16 µM in FRS2-FLAG (Fig. 6e). Thus, increasing FRS2 protein levels lead to an increased amplitude of response to bFGF treatment. Importantly, however, this increased response to bFGF is efficiently blocked by **7** at the IC50 concentration calculated for wt cells (Fig. 6e and f). Conversely, DAOY cells with reduced FRS2 expression invaded the collagen I matrix considerably less efficiently compared to control cells (Fig. 6g). The calculated IC50 in DAOY cells with reduced FRS2 was increased by more than 2.5 fold to 7.96 µM compared to control (3.01 µM), strongly suggesting that compound **7** effects are mediated via FRS2 (Fig. 6e). Consistently, we also observed nearly no reduction in invasion in siFRS2 cells around the IC50 concentration of control cells (Fig. 6i).

**Fig. 7:**
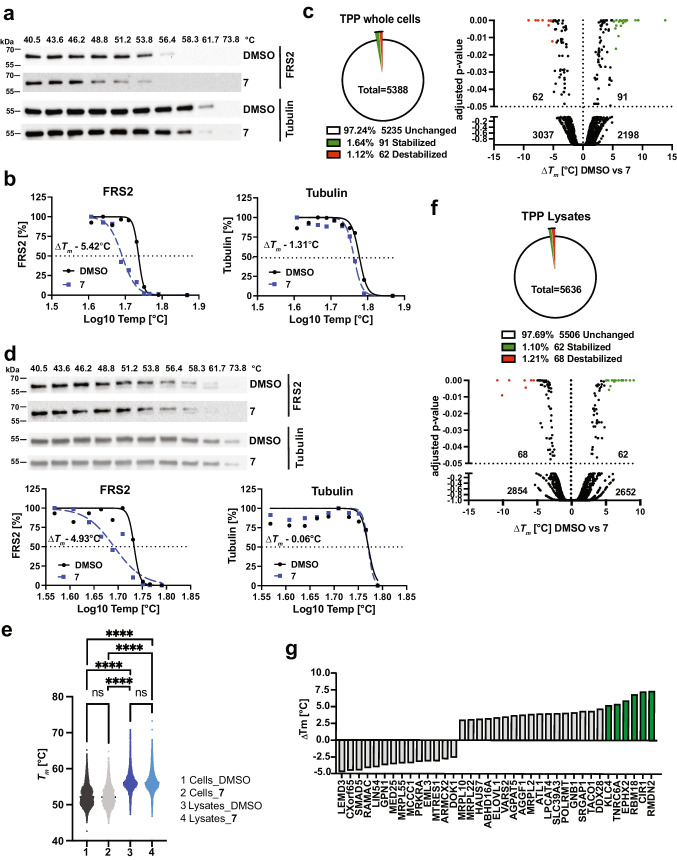
On/off target activity of compound **7** in cells and lysates. **a**) IB of FRS2 and tubulin from whole cell CETSA after DMSO or compound **7** treatments. **b**) Nonlinear fit of FRS2 and tubulin abundance in CETSA from DMSO- or compound **7**-treated whole cells shown in a. **c**) Left: Pie chart of percentage stabilized and destabilized proteins (p < 0.05) from whole cell TPP analysis. Right: Volcano plot of *∆T*_*m*_ from proteins of which high quality melting curves were obtained in both conditions. Red dots ∆*T*_*m*_ ≥ -5 °C, green dots: ∆*T*_*m*_ ≥ 5 °C. **d**) Upper: IB of FRS2 and tubulin in cell lysate CETSA after DMSO or compound treatment. Lower: Nonlinear fit of FRS2 and tubulin abundance of CETSA from DMSO or **7**-treated cell lysates. **e**) Scatterplot dot plot of *T*_*m*_ distributions of all proteins with high quality melting curves of whole cell and lysate TPP analyses. **f**) Upper: Pie chart of percentage stabilized and destabilized proteins (p < 0.05) from lysate TPP analysis. Lower: Volcano plot of *∆T*_*m*_ from proteins of which high quality melting curves were obtained in both conditions. Red dots ∆*T*_*m*_ ≥ -5 °C, green dots: ∆*T*_*m*_ ≥ 5 °C. **g**) Bar plot with gene symbols of proteins with significantly altered *T*_*m*_ (p < 0.05) after manual inspection of melting curves. Green bars: ∆*T*_*m*_ ≥ 5 °C

To further confirm on-target activity of compound **7** in cells, we used the cellular thermal shift assay (CETSA [36]) in DAOY cells, which interrogates protein stability across a temperature gradient. We monitored the abundance of soluble FRS2 in the absence and presence of 10 µM compound **7** over a range of ten temperatures between 40.5 and 73.8 °C by IB (Fig. 7a). We observed a negative shift in the correspondingly calculated melting temperature (*T*_m_) curve of FRS2 in the presence of **18.7** with Δ*T*_m_ between -5.4 and -4.93 °C. Under the same conditions, the negative control protein beta-tubulin displayed a weak shift with a Δ*T*_m_ between -1.31 and -0.06 °C (Figs. 7b, S9A).

To explore potential off-target activities of compound **7**, we performed thermal proteome profiling (TPP) [37] in intact DAOY cells. We identified approximately 8000 proteins in ~ 6750 protein groups. For ~ 5400 proteins, we succeeded to fit high-quality melting curves (R^2^ > 0.8 in both conditions) that passed the chosen filter criteria (plateau < 0.3 in vehicle group). For these proteins, *T*_m_ differences between DMSO and compound **7** treatments and adjusted *P* values were determined (Supplementary Table 4). 153 candidates showed a significant *T*_m_ difference with an adjusted *P* value of < 0.05, of which 91 proteins (1.64%) were stabilized and 62 (1.12%) destabilized by compound **7** treatment (Fig. 7c). The corresponding *T*_m_ differences were distributed between – 9 and + 13 °C (Fig. S6b). Altered *T*_m_ in proteins after compound **7** treatment could be the result of direct binding or indirect downstream effects of compound-stabilized or -destabilized proteins [38]. We therefore also determined *T*_m_ of FRS2 and tubulin in lysates of DAOY cells treated either with DMSO or 10 µM compound **7**. We again observed a negative shift in the *T*_m_ curve of FRS2 in the presence of compound **7**, with a DΔ*T*_m_ of -4.93 °C. The calculated Δ*T*_m_ of tubulin in lysates was 0.64 °C (Fig. 7d). We next explored potential direct off-target molecules of compound **7**, by comparing DΔ*T*_m_ between DMSO- or compound **7**-treated cell lysates using TPP. Independent of the treatment, the average *T*_m_ in lysates was increased by approximately 4 °C compared to whole cells (Fig. 7e), which is consistent with previous reports [38, 39]. We succeeded to fit high quality melting curves for 5638 proteins. 62 proteins (1.1%) were stabilized and 68 (1.21%) destabilized by compound **7** treatment (*P* value < 0.05) (Fig. 7f, supplementary Table 5). A total of 35 (0.65%) proteins in intact cells and 24 (0.44%) proteins in the lysate displayed a *T*_m_ shift of equal or more than 5 °C (Fig. S9c). We manually inspected the melting curves, removed curves, where single outliers altered slope and shape of the curve disproportionally and generated a small list of high-probability interactors (Fig. 7c). We found no overlap in Δ*T*_m_ between total cells and lysates, and—probably due to low abundance—we were not able to detect endogenous FRS2 protein in neither of the two TPP approaches.

From these experiments we concluded that compound **7** interacts with its target protein FRS2 in whole cells. However, similar *T*_m_ shifts in the lysate proteome reveal the interaction of compound **7** with additional proteins and indicate potential functional off-target activities to be further investigated.

## Discussion

The identification of novel potential drug targets and resistance mechanisms for known anticancer drugs have strongly increased the demand for new drugs, and in particular for drugs against so-far unexploited targets. The aim of this study was to explore whether modulation of FRS2 function by small molecule compound binding is a successful strategy to interfere with FGFR-driven oncogenic functions of cancer cells. To this end we have identified novel small-molecule inhibitors of FGFR signaling using an in silico discovery approach that enabled the identification of ligands of the PTB domain of FRS2. We confirmed direct compound-FRS2 interaction by biophysically assessing compound impact on thermal stability of the PTB domain of FRS2 and by NMR. We functionally validated anti-tumor compound activities in human cancer cells in vitro and in a tumor model of ovarian cancer in vivo. Using a proteome-wide compound-target interaction analysis, we furthermore identified potential off-targets of the lead compound that will guide further drug development efforts.

Three chemically distinct compounds were identified that repress FGFR-dependent signaling and cancer cell invasiveness. One of these, compound **7** showed potent FGFR pathway inhibition in several cell models in vitro and anti-tumor activity in a human cancer model in vivo. Bioactivity of **7** has not been reported so far, while the structural analog **10** was described as an inhibitor of microbes, parasites and cancer targets, however, not against FGFR-related proteins (https://pubchem.ncbi.nlm.nih.gov/compound/3406013). Our biophysical data indicate a destabilizing effect of **7** upon binding to FRS2, whereas the cognate ligand FGFR_PEP, a peptide corresponding to the natural ligand sequence of FGFR1, is stabilizing. This could indicate that **7** interferes with FRS2 functions by disturbing its functional interaction with the FGFR and thereby impairing FGFR-dependent signal transmission. The inhibitory effects of **7** in FGFR signaling are supported by the observed reduction of acute FRS2_Y436_ phosphorylation in bFGF-stimulated cells and impaired activation of the MAPK pathway effector ERK. The destabilization effect of **7** in vitro was still strongly present even after co-incubation with FGFR_PEP and despite the almost 100-fold stronger affinity of the peptide. Importantly, compound effects phenocopy depletion of FRS2 by siRNA, both in collagen invasion and transwell migration as well as in ERK phosphorylation. Increasing FRS2 expression also leads to an increase of the amplitude of bFGF-induced invasiveness. Compound **7** effectively blocks this increase, whereas invasiveness of FRS2-depleted cells is not affected. Combined, these observations strongly argue for a direct inhibitory effect of compound **7** acting on FRS2 and repressing its pro-invasive functions.

At present, the compound affinities of 60–140 μM are weak, as expected from small molecules in early screening hits [40], requiring further medicinal chemistry modifications to increase affinity. Interestingly, NMR-derived CSPs indicate that compounds **3.14**, and the bioisosteres of **7**, **13** and **14,** bound to a site that is occluded by the natural FGFR ligand peptide, and which was therefore not targeted during virtual screening. Accordingly, the detected CSPs are small, as expected if the compound is competing with the peptide for FRS2 binding. Nevertheless, we observed protein-compound interactions that are typically observed in drug-receptor interactions such as π-stacking or hydrogen bonds. Inspection of the protein:compound complexes reveals that the pocket is only partially filled, indicating that a fragment-growth strategy could substantially improve their binding affinity in a hit-to-lead drug development effort.

Our aim was to achieve repression of FGFR-driven tumor growth and progression through interfering with FRS2 function as an alternative targeting strategy. FGFR alterations are not confined to one tumor entity e.g. high or low grade or adult or pediatric, which renders the assignment of FGFR alterations as tumor driving lesions more challenging [41]. Besides the numerous FGFR alterations in adult cancers [9], some alterations are strongly associated with pediatric low grade neuroepithelial lesions such as FGFR1 duplication, and FGFR1/3-TACC1 or FGFR2-CTNNA3 fusions [41] may be considered as hallmarks of these tumors. We found that FRS2 depletion or compound **7** treatment repressed bFGF-induced invasiveness in the FGFR3-TACC1 fusion-driven RT-112 cells, indicating that an FRS2-targeting strategy may be successful in tumors with these lesions as well. ERK is necessary for bFGF-induced invasiveness in DAOY cells [4]. Although **7** reduces FRS2_Y436_ and ERK_Thr202/Tyr204_ phosphorylation in vitro, we were not able to correlate the phosphorylation of these residues with repressed tumor growth in vivo in SKOV3 cells. It is possible that the still detectable levels of FRS2_Y436_ phosphorylation in compound **7**- or BGJ398-treated animals were the result of a re-bound effect during or towards the end of the treatment period. Consistent with this possibility is the increased tumor growth in compound **7**-treated animals towards the end of the treatment period. Interestingly, tumors of animals treated with BGJ398 showed similar growth dynamics, suggesting that emerging resistance is rather related to FGFR pathway inhibition in this context and not to the specific mode of action of **7**. A similar phenomenon of tumor re-growth after a lag-phase was also observed in AGS tumors, both in compound **7** and in BGJ398-treated animals.

FRS2 may also be involved in alternative signaling pathways to promote tumor growth. In prostate cancer [42], FRS2 signaling promotes tumor angiogenesis, and FRS2 overexpression by gene duplication in bladder cancer is associated with tumor vascularization and poor prognosis [23]. Thus, tumor growth repression by **7** in the SKOV3 model could also be a consequence of decreased or delayed neovascularization. An FRS2 binding site was also discovered in the oncogenic Anaplastic Lymphoma Kinase (ALK), which is necessary for the transforming activity of ALK [43]. ALK is overexpressed in ovarian cancer and associated with an aggressive, metastatic phenotype [44, 45]. Finally, FRS2 inhibition could also be suitable as a combinatorial therapy strategy in KRAS-driven tumors with acquired MEKi resistance, which are sensitive to FRS2 depletion [46], or to bypass FGFR-driven resistance to epidermal growth factor receptor (EGFR) [47] or mesenchymal epithelial transition (MET) inhibition [48].

Using kinome-wide thermal proteome profiling (TPP), we identified several proteins with compound **7**-dependent increased or decreased thermal stability. Some of the thermal shifts observed are equal or greater to what we observed for FRS2. These proteins must be considered as potential off-targets of **7**. None of the proteins with altered melting temperature is a known regulator of FGFR signaling, making it unlikely that the effects of **7** we observed on bFGF-induced signaling are the consequence of the off-target activities. Nevertheless, the discovered off-targets and potentially associated, non-specific toxicities must be reduced in subsequent lead development efforts. We also observed that 3/12 tumors did not display reduced growth in compound **7**-treated animals in vivo for which the underlying causes remain unknown. Further pre-clinical investigations in cell and tissue models are required for better understanding the pharmacokinetic and efficacy characteristics of **7**.

To conclude, we provide strong evidence that compound **7** interference with FRS2 functions, represses oncogenic FGFR signaling and invasiveness in human cancer cells and can reduce tumor growth in vivo. Our study thus warrants further pre-clinical development of **7** for the treatment of human cancers with activated FGFR signaling and oncogenesis driven by the overexpression of FRS2.

## Materials and methods

### Experimental design

The objective of this study was to specifically target the adaptor protein FRS2, which transmits oncogenic FGFR signaling to downstream effectors in various human cancers. Our hypothesis was that by impairing the functional interaction between the FGFR and FRS2, we can block receptor-derived signal transmission. We applied a pharmacophore-based computational screening approach to identify small molecules predicted to bind the PTB domain of FRS2, which mediates the interaction between the receptor and the adaptor protein. We used the spheroid invasion assay with a well-established sensor cell line to measure compound effects on the capability of cells to invade a 3D matrix, and we selected compounds with the desired functional activity (repression of FGFR-driven invasiveness). We confirmed the binding of selected molecules to the PTB domain using biophysical assays and determined the location of compound binding effects in the targeted PTB domain by chemical shift perturbation analysis using nucleic magnetic resonance. We confirmed the inhibition of FGFR-driven invasiveness and pathway activation in several established human cancer models with described aberrations in FGFR signaling. Based on the combined analysis of the data from the biophysical and functional analyses with an in vivo pharmacokinetic study, we selected a lead compound. The effect of the lead compound on tumor growth was assessed in vivo using established flank models for ovarian and colon cancer. By using thermal stability assays at single protein and proteome level in intact cells and in cell lysates, we confirmed on target and determined putative off-target activities of the lead compound.

### Cell lines used

DAOY human MB cells were purchased from the American Type Culture Collection (ATCC, Rockville, MD, USA). DAOY cells were cultured as described in [4]. Cell line authentication and cross contamination testing was performed by Multiplexion by single nucleotide polymorphism (SNP) profiling. ONS-76 cells were generously provided by Michael Taylor (SickKids, Canada). RT-112 (ACC418) was purchased from the Leibnitz-Institut DSMZ (Braunschweig, Germany). AGS (ATCC CRL-1739), DMS114 (ATCC CRL-2066), HCT116 (ATCC CCL-247), M059K (ATCC CRL-2365), SKOV3 (ATCC HTB-77), SNU-16 (ATCC® CRL5974) and SW 780 (ATCC CRL-2169) were purchased from LGC Standards GmbH, Wesel, Germany) and cultured according to LGC instructions.

### Spheroid invasion assay (SIA) and automated cell dissemination counter (aCDc)

1000—2500 cells/well in 100 μl were seeded in 96 well Corning® Spheroid microplate (CLS4520, Sigma-Aldrich) or in cell-repellent 96 well microplate (650,790, Greiner Bio-one). SIA and analysis was performed as described before [32]. In brief: After spheroid formation (24 – 72 h after seeding), 70 µl medium was removed and replaced with the collagen mixture (2.5 mg/ml Pure Col Collagen I (Advanced Biomatrix)), DMEM 1x (from 10 × stock, Sigma, D2429) and 0.4% Sodium bicarbonate (Sigma, S8761)), resulting in a final collagen concentration of 1.75 mg/ml. Embedded spheroids were stimulated with bFGF (100 ng/ml if not otherwise stated), HGF (30 ng /ml) or EGF (20 ng/ml) and distance of invaded cells quantified 24 – 48 h after embedding. Compounds and BGJ398 were added in Dimethylsulfoxid (DMSO) and corresponding DMSO concentrations were used in control samples.

### siRNA transfections for SIA

siFRS2 in RT-112 cells: 60,000 RT-112 cells/well in 2 ml medium were seeded in 6 well plates and incubated at 37 °C, 5% CO_2_. The following day, cells were transfected with 10 nM siRNA using Lipofectamine RNAiMAX Transfection Reagent (13,778,075; Invitrogen) and Opti-MEM® medium (15,392,402; Gibco). After 6 h, the cells were harvested and further processed for the SIA protocol.

siFRS2 in DAOY cells: 50,000 cells/well in 2 ml medium were seeded in 6 well plates and incubated at 37 °C, 5% CO_2_. The following day cells were transfected with 10 nM siRNA (5 nM Silencer Select, 5 nM Stealth RNAi siRNA) using Lipofectamin RNAiMAX Transfection Reagent (13,778,075; Invitrogen) and Opti-MEM® medium (15,392,402; Gibco). After 6 h, the medium was exchanged with fresh complete medium and further processed for the SIA protocol.

### Generation of FRS2-FLAG overexpressing DAOY

Lentiviral construct pLV[Exp]-hPGK > hFRS2[NM_001278357.1](ns)*/3xFLAG:P2A:Puro was ordered from VectorBuilder (Santa Clara, CA, USA). A FLAG-tag (DYKDDDDK) was fused to the C-terminus of FRS2. Lentiviral particles were produced by transfection of HEK-293 T cells with transfer plasmids, psPAX2 (#12,260, Addgene) and pCMV-VSV-G plasmid (#8454, Addgene) in a ratio of 5:3:2 using polyethlenimine (24,765–2. Polysciences). Virus supernatant was harvested 30 h post transfection and filtered. DAOY cells were incubated 24 h with supernatant containing viral particles. Two days after transduction, cells were selected with puromycin (2 µg/µl). For SIA, the DAOY FRS2-FLAG cell line was processed according to the SIA protocol.

### Boyden chamber assay

Transwells with 5 μm pore size (83.3932.500; Sarstedt) were coated with 50 µl of 0.06 μg/μl Pure Col Collagen I (5005; Advanced BioMatrix) dissolved in 100% EtOH for a final concentration of 10 μg/cm^2^. 10,000 – 15,000 cells were resuspended in serum-free medium or serum-free medium containing the appropriate treatment and seeded in the upper chamber of a 24 well plate containing the coated transwell. The lower chamber was filled with either serum-free medium or serum-free medium with bFGF (25 ng/ml). Transwell migration was allowed for 24 h (DMS114) or 48 h (HCT116) at 37 °C, 5% CO_2_. Inserts were washed twice with PBS and the remaining non-invading cells on the upper surface of the membrane were removed using a cotton swab. The cells were fixed in 4% paraformaldehyde (28908; Thermo Fisher Scientific) in PBS for 5 min at RT. Fixed cells were washed twice with PBS and stained with DAPI for 15 min at RT. Inserts were washed three times in PBS before images of DAPI-stained nuclei were acquired using a widefield Nikon Ti2 microscope at 40 × magnification. Nuclei were counted on the membrane areas using ImageJ software and plotted on Prism 9 software (GraphPad).

### 3D-Cell viability (Cell TiterGlo) assay

Cell viability was determined using CellTiter-Glo® 2D or 3D cell viability assays (#G9242, #G9682, Promega). 500 cells/25 µl were seeded in flat bottom (#781,091, Greiner bio-one) or U-low adhesion (#4516, Corning) 384-well plate, 24 h prior to treatment. Increasing concentrations of compounds are deposited on cells using a HP Digital Drug Dispenser with DMSO total volume normalization. After 48 h, the CellTiter-Glo® 2D or 3D reagent was added (volume/volume) following manufacturer’s instructions. Plates were incubated at RT (room temperature) under agitation for 30 min and luminescence representing the number of viable cells was quantified with a Cytation 3 imaging reader (BioTek®). Experiments were performed independently three times with three technical replicas each.

### Immunoblotting (IB)

Cells were serum starved o.n. and 10 µM compound or 1 µM (BGJ398) were added 3 h before stimulation with 100 ng/ml bFGF for 15 min. Cells were lysed in RIPA buffer containing protease inhibitor cocktail and lysates resolved by SDS-PAGE. Membranes were probed with primary antibodies against phospho-FRS2, FRS2, phospho-ERK1/2, ERK1/2, pAKT, AKT and tubulin. Integrated density of immuno-reactive bands was quantified using Adobe Photoshop CS3. Integrated densities of phospho bands relative to non-phospho bands of same protein were calculated and plotted as fold-change relative to untreated control conditions. Compounds and BGJ398 were added in DMSO and corresponding DMSO concentrations were used in control samples.

### RNA expression analysis by RT-qPCR

Cells were seeded in 6-well plate for 24 h to reach 80–90% confluency the following day. RNAs were extracted using the RNAeasy® plus mini kit (#74,136, Qiagen) according to manufacturer’s instructions. 150 ng RNA were used for reverse transcription in 20 µl reaction containing RNAse inhibitor with the high-capacity cDNA reverse transcription kit (#4,374,967, Applied Biosystems, ThermoFisher Scientific). qPCR was performed on cDNA with the TaqMan™ gene expression master mix (#4,369,016, ThermoFisher Scientific) using a 7900 HT fast real-time PCR system (Applied Biosystems). The relative expression level represented by the relative cDNA level was determined according to the standard curve method with a reference sample. Experiments were performed independently three times with two technical replicas.

### Chemical libraries

#### F-Series library

Collection of commercially available fast-delivery compounds of the suppliers Asinex Corp., ChemBridge Corp., ChemDiv, Enamine, Specs, UkrOrgSyntez Ltd. And Vitas-M Laboratory Ltd.

E compound series library:

- PrestwickChemicalLibrary: Collection of 1280 FDA-approved compounds.
- NCCR54k: Collection of around 54 k compounds intended to represent the commercially-available chemical space.
- ChemicalDversityExtension: Collection of around 14 k compounds that extends NCCR54k with molecules that were initially excluded for various reasons (e.g. molecular weight, number of stereo-centers)
- PPI: Collection of around 5 k compounds targeted at disrupting protein–protein interactions.
- NaturalProducts: Collection of around 2.5 k commercially available natural compounds. These compounds are enantiomerically pure but their stereo chemistry is not always known. Therefore, several of them appear to have identical structure (because of the unspecified stereocenters) but the suppliers guarantee that different catalog IDs correspond to different compounds.

### In silico screening methods

Relevant PPIs were analyzed by iPRED analysis based on the only known structure of the FRS2-PTB 1XR0. Calculations were performed via the online available iPRED-tool. Virtual screening was performed with LigandScout based on PDB 1XR0. The co-crystallized peptide was removed, the surface analyzed for pockets by the in-built pocket calculator. Two shallow pockets the first formed by the alpha-helix (A94-M105) and the beta-sheet (L33-L47), the second being complementary to the FGFR-peptide sequence I_pep_13-R_pep_16 (FGFR_PEP: HSQMAVHKLAKSIPLRRQVTVS), were identified and isolated. Amino acids were charged at pH 7 according to their pK_A_ and energy minimized. Apo Site grids were calculated with a maximum of 4 H-bond acceptors, 3 H-bond donors, 2 positively ionizable groups, 1 negatively ionizable group, 2 aromatic groups and 4 hydrophobic moieties. Accessibility was considered in the calculation. The generated pharmacophore was run against a database containing all fast-delivery compounds of the suppliers Asinex Corp., ChemBridge Corp., ChemDiv, Enamine, Specs, UkrOrgSyntez Ltd. And Vitas-M Laboratory Ltd. with an overall compound number of 3.5 million (F-series) and against a collection of compounds from libraries listed above (E-series). Screening was performed under the Pharmacophore Fit-model with maximally 4 pharmacophoric features being omittable in order to be considered. Hits from both pockets were then combined, ranked by the provided score and selected based upon chemical diversity. Fragment-based screening and docking was performed with LigandScout vers. 4.2.1 (Inte:Ligand, Vienna, Austria) and data analyzed with Microsoft Office Excel 365 (Microsoft Corp., Seattle, USA).

### Nano-Differential Scanning Fluorimetry (nanoDSF)

Assay establishment and pilot nanoDSF experiments were performed with a Tycho® system. Each experiment was performed by mixing the protein and ligand of interest at a 1:1 ratio and subsequent incubation for 15–30 min at room temperature. Controls of protein alone and ligand alone in identical buffer compositions as for the complex were measured accordingly. The temperature gradient ranged from 35 to 95 °C, and the heating rate was 30 °C/min. Inflection temperatures TI were calculated from the first derivative of the spectra obtained at the ratio of the signals at 350 nm to those at 330 nm. ΔTI of a complex was calculated as following: Δ*TI,complex* = *TI,complex – TI,protein.*

The in-built software version 1.1.5.668 (NanoTemper Technologies, Munich, Germany) and Microsoft Office Excel 365 (Microsoft Corp., Seattle, USA) was used for data analysis. Data was plotted with GraphPad Prism vers. 8.3.0 (GraphPad Software, San Diego, USA).

Purified FRS2_PTB or GB1_FRS2_PTB protein tagged with 6 × Histidine residues and the B1 immunoglobulin binding domain of the streptococcal protein G (GB1) were diluted in the protein buffer (100 mM sodium phosphate, 50 mM NaCl, 0.5 mM EDTA, 50 mM arginine, 1 mM TCEP, pH 7.0) to 30 μM, GB-1 to 40 μM. FGFR_PEP, compounds or paracetamol (PARA) as negative control were dissolved at 1 mM in the protein buffer supplemented with 10% DMSO. Compound and protein were mixed at 1:1 volume ratio yielding a final concentration of 15 μM and 20 μM for FRS2_PTB/GB1_FRS2_PTB and GB1, respectively, as well as 500 μM for FGFR_PEP or compounds. The mixture was incubated for 15 min. Each compound was measured in triplicates. Protein controls were measured in six replicates in the beginning. The quality of the screen was assessed by the Z-factor. nanoDSF validation studies were performed on a Prometheus® system in high sensitivity capillaries. Samples were heated with 1 °C/min from 20 to 95 °C. Each compound was measured in triplicates. GB1-FRS2_PTB and GB1 were diluted in the protein buffer (100 mM sodium phosphate, 50 mM NaCl, 0.5 mM EDTA, 50 mM arginine, 1 mM TCEP, pH 7.0) to 30 μM. Compounds **3.3**, **3.4**, **3.18** as well as the **3.18** analogues were dissolved in 100% DMSO at 50 or 100 mM and further diluted to 1 mM with a final DMSO-concentration of 100%. FGFR_PEP was dissolved at 1 mM in the protein buffer supplemented with 10% DMSO. Compound and protein were mixed at 1:1 volume ratio yielding final concentrations of 15 μM protein and 500 μM for the compounds. FGFR_PEP was measured against both GB1-FRS2_PTB and GB1, all other compounds only against GB1-FRS2_PTB. The mixture was incubated for 15 min before measurement. The data was analyzed for both the ratio of signals at 330 and 350 nm and at 350 nm alone. For the competition assays, the PTB domain and the FGFR peptide were mixed at a 1:5 ratio, and ligand competition was assayed in the presence of 500 μM compound.

### Microscale thermophoresis (MST)

Protein labeling for MST was performed with the 2nd Generation BLUE-NHS dye. GB1-FRS2_PTB- was labelled at 20 μM with 60 μM dye and incubated for 30 min in the dark at room temperature. The labelling was performed in the protein buffer without arginine supplementation. It was rebuffered to protein buffer (100 mM sodium phosphate, 50 mM NaCl, 0.5 mM EDTA, 50 mM arginine, 1 mM TCEP, pH 7.0). The dye was subsequently removed by gravitational flow chromatography and the protein concentration determined by UV-spectroscopy.

The assay was established with the FGFR_PEP. The peptide was dissolved in protein buffer with and without 10% DMSO supplementation in a 1:1 serial dilution from 1 mM to 61.04 nM. A total volume of 10 μl of 50 nM labelled protein was added to 10 μl of the peptide dilution series for a final labelled protein concentration of 25 nM. The samples were incubated for 15 min at RT. Premium-coated capillaries were used, excitation power was set at 20%, MST-power to 40% (4 K temperature gradient) with a laser-on time of 20 s and a laser-off time of 3 s. Temperature was set to 25 °C. Each measurement was repeated twice. The interaction was measured in duplicates for the non-DMSO-supplemented buffer and in triplicates for DMSO-supplemented buffer. The compounds **3.3**, **3.14**, **3.18** as well as the analogs **18.2**, **18.7**, **18.8** and **18.9** were dissolved in 100% at 50 or 100 mM and further diluted to 1 mM with a final DMSO-concentration of 100%. The compounds were diluted in a 1:1 serial dilution from 1 mM to 61.04 nM in protein buffer supplemented with 10% DMSO. 10 μl of 50 nM labeled protein was added to 10 μl of each compound dilution for a final labeled protein concentration of 25 nM and a DMSO-concentration of 5%. The samples were incubated for 15 min, and MST assessed as described above. Each measurement was repeated twice. The interaction was measured in two independent duplicates. LIFC was observed in all measurements. Subsequently, 10 μl of compounds **3.3**, **3.14** and **3.18** at 2 mM, 1 mM, 500 and 250 μM were incubated with 10 μl of 50 nM dye to yield final compound concentrations from 1 mM to 125 μM in a 1:1 dilution series and 25 nM dye concentration. The mixture was then measured for their initial fluorescence once.

MST-experiments were performed with MO.Control vers. 1.6 (NanoTemper Technologies, Munich, Germany) and data analyzed with MO.Analysis vers. 2.3 (NanoTemper Technologies, Munich, Germany). Data was plotted with GraphPad Prism vers. 8.3.0 (GraphPad Software, San Diego, USA).

### Expression and purification

The PTB domain of human FRS2, residues 15 to 135, were cloned into a pEM3BT2 vector as a fusion with an N-terminal 6 × histidine tag, GB1 with or without FGFR1 peptide (FGFR_PEP HSQMAVHKLAKSIPLRRQVTVS) and was then used to transform BL21 (DE3) cells for plasmid DNA amplification and protein expression. To induce protein expression, the transformed clones were cultured at 37 °C in labelled M9 media supplemented with 100 µg/ml of ampicillin using ^15^ N-NH_4_Cl and ^13^C-glucose as the sole sources of ^15^ N and ^13^C. When the culture reached an OD_600_ of 0.6 to 0.8, protein expression was induced by adding 1 mM of IPTG at 25 °C overnight.

The bacterial cells were collected by centrifugation at 5000 × g for 15 min and the pellets lysed by adding 10 mg/ml of lysozyme and sonication. The lysates were then centrifuged for 30 min at 18,000 rpm at 4 °C. The supernatants were filtered using a 0.2 µm membrane and passed on an equilibrated, 5 ml Ni–NTA column using ÄKTA prime at 4 °C. The protein/resin complex was then washed with 5 column volumes each using low salt wash buffer and high salt wash buffer. The protein was eluted from the resin using the elution buffer containing 100 mM Tris, 50 mM NaCl and 500 mM (pH 7.0) imidazole. Following SDS-PAGE analysis of the eluted fractions, the protein was dialyzed overnight against the buffer containing 50 mM phosphate buffer, 50 mM NaCl, 1 mM TCEP and 0.5 mM EDTA, pH 7.0 at 4 °C. The protein fractions were then pooled and concentrated to 250 µM using Amicon Ultra – 4 (10 KDa cutoff) centrifugal filters. The final protein concentration was determined by measuring absorbance at 280 nm using a nanodrop instrument and further used for NMR analysis.

### NMR spectroscopy

NMR experiments were recorded using 250 µM solutions of proteins in 50 mM phosphate buffer, 50 mM NaCl, 1 mM TCEP and 0.5 mM EDTA, pH 7.0. All NMR experiments were recorded at 310 K on Bruker Avance-Neo 600 or 700 MHz spectrometers equipped with cryoprobes. For backbone assignment of the GB1-FRS2_PTB_FGFR_PEP fusion protein a ([79% 2H, 99% [^13^C,^15^ N])-labeled sample was used. Experiments were selected from the Bruker standard pulse sequence library. [^15^ N,^1^H]-HSQC experiments were of the sensitivity-enhance type [49] and comprised spectral widths of 14(F2, ^1^H) and 36 (F1, ^15^ N) ppm with 1024*128 complex data points. HNCACB / HN(CO)CACB spectra were used to link sequential amide groups via Cα/Cβ resonances. In addition, sequential connection was confirmed via common carbonyl resonances using the HNCO and HN(CA)CO experiments. The usage of the HN(CACO)NH experiment proved to be particularly useful by establishing sequential contacts in cases where the Cα/Cβ correlations were missing by directly establishing sequential ^15^ N connectivity. All spectra were processed in TOPSPIN using linear prediction and spectra were analyzed in the program CARA.

STD and WaterLOGSY experiments were measured at 7ºC on a Bruker AvNEO 600 spectrometer equipped with a TCI cryoprobe. In the STD experiments saturation was achieved by applying a series of Gaussian-shaped pulses at a RF field strength of 54 Hz (max.) for 3 s at -0.5 ppm (-40 ppm for the reference experiment) and 512 scans were accumulated. The WaterLOGSY was measured with 256 scans and a mixing time of 1.5 s. A 7.5 ms Gaussian-shaped 180° pulse was used to control the water magnetization. For both experiments samples of 25 µM GB1-FRS2_PTB with a 20-fold excess of ligand (500 µM) were used or with the ligand alone for the control experiment.

### Thermal Protein Profiling (TPP) in intact cells

Two 15 cm dishes with 0.5 × 10^6^ DAOY cells per dish were grown in full medium o.n. Prior to compound treatment, cells were washed once in PBS. Complete growth medium containing 10 µM compound was then added and cells incubated for another 90 min at regular culture conditions. Medium was removed, cells washed with PBS containing 10 µM compound and detached using trypsin containing 10 µM compound. Trypsin was neutralized using regular growth medium containing 10 µM compound and cells were collected by centrifugation (300 × g, 25 °C, 5 min). After one more wash in PBS containing 10 µM compound, cell pellet was resuspended in 220 µl PBS containing 10 µM compound. Ten 20 µl aliquots were dispensed in a pre-cooled PCR plate, which was sealed with aluminum foil and placed on ice until heat treatments. Heat treatment was performed in a gradient PCR machine. The following temperatures were used: 40.5, 43.6, 46.2, 48.8. 51.2, 53.8, 56.4, 58.3, 61.7 and 73.8 °C for three min. Plate with samples was kept at RT for 3 min and then kept on ice until centrifugation for 2 min at 500 g at RT.

Cells were lysed by adding 30 µl TPP lysis buffer (PBS, 1.33% IGEPAL, 1.66 mM MgCl_2_, 1.66 × cOmplete protease Inhibitors, 1.66 × PhosStop, 416.6 U/ml benzonase) per sample, resulting in a final concentration of 0.8% IPEGAL, 1 mM MgCl_2_, 1 × cOmplete protease Inhibitors, 1 × PhosStop, 250 U/ml benzonase. Samples were incubated 1 h at 4 °C, with shaking at 500 rpm. Samples are filtered using a MultiScreenHTS HV-Filterplate (Merck, MSHVN4510). Membrane wells were prewetted with 50 µl PBS and centrifuged at 2000 × g, RT, 3 min. The lysates were centrifuged at 2000 g, RT, 3 min and 40 µl of each supernatant was transferred to the filter plate and centrifuged at 500 g at RT for 5 min. Flow-through of samples was collected in a fresh 96-well plate and kept on ice. 20 µl of samples was snap-frozen and stored at -80 °C for further analysis by mass-spectrometry. 5 µl was used for protein concentration determination using BCA assay.

### TPP in lysates

Four 15 cm dishes with 0.5 × 10^6^ DAOY cells per dish were grown in full medium o.n. Medium was then removed, cells washed with PBS, detached using trypsin, collected in regular growth medium, centrifuged (300 g, 25 °C, 5 min), washed with PBS and resuspended in 550 µl ice-cold PBS. Cells were lysed with three freeze–thaw cycles (freeze in liquid N_2_, thaw in 25 °C heat block, vortex quickly) and lysates were placed in aliquots of 250 µl on ice. Compound or solvent was added to 10 µM final concentration and samples were incubated 20 min at 4 °C under rotation. Ten 20 µl aliquots were dispensed in a pre-cooled PCR plate, which was sealed with aluminum foil and placed on ice until heat treatments. Heat treatment was performed in a gradient PCR machine with the following temperatures 40.5, 43.6, 46.2, 48.8. 51.2, 53.8, 56.4, 58.3, 61.7 and 73.8 °C for three min. Plate with samples was kept at RT for 3 min and then kept on ice until centrifugation for 2 min at 500 × g at RT.

The soluble fraction of the lysates was recovered by addition of 30 µl TPP lysis buffer (PBS, 1.33% IGEPAL, 1.66 mM MgCl_2_, 1.66 × cOmplete protease Inhibitors, 1.66 × PhosStop, 416.6 U/ml benzonase) per sample, resulting in a final concentration of 0.8% IPEGAL, 1 mM MgCl_2_, 1 × cOmplete protease Inhibitors, 1 × PhosStop, 250 U/ml benzonase. Samples were incubated 1 h at 4 °C, with shaking at 500 rpm. Samples are filtered using a MultiScreenHTS HV-Filterplate (Merck, MSHVN4510). Membrane wells were prewetted with 50 µl PBS and centrifuged at 2000 g, RT, 3 min. The lysates were centrifuged at 2000 g, RT, 3 min and 40 µl of each supernatant was transferred to the filterplate and centrifuged at 500 g at RT for 5 min. Flow-through of samples was collected in a fresh 96-well plate and kept on ice. 20 µl of samples was snap-frozen and stored at -80 °C for further analysis by mass-spectrometry. 5 µl was used for protein concentration determination using BCA assay.

### Cellular thermal shift assay (CETSA) using IB

For a cellular thermal shift assay (CETSA) run, 7.5 µl of the flowthrough samples of the TPP preparation was mixed with 3 µl 4 × Laemmli buffer containing 50 mM DTT and boiled for 5 min at 95 °C. 10 µl of each sample was assayed by immunoblot using antibodies against FRS2 and beta-tubulin.

### Mass spectrometry – ESI MS

Sample preparation: Protein samples were processed using a modified SP3 clean-up and digestion procedure on a KingFisher Flex (Thermo). In short, protein samples were reduced/alkylated using 2 mM TCEP, 15.6 mM CAA for 30 min at 60 °C. Subsequently, samples were adjusted to 50% ethanol and bound to 100 μg magnetic beads. Bound proteins were washed three times with 80% ethanol and on-bead digested overnight in 50 mM TEAB including Trypsin/Lys-C (1:50, enzyme:protein) at 37 °C. Resulting peptides were recovered and dried to completeness.

For isobaric labelling, peptides were resuspended in 20 µl 50 mM TEAB and 0.1 mg TMT reagent was added in 5 µl anhydrous ACN and incubate for 1 h at room temperature with shaking. Unused labelling reagent was quenched by adding 1.25 µl of 5% hydroxylamine solution and incubation for 15 min at RT. Quenched samples were pooled and cleaned using C18 solid phase extraction and subsequently dried down.

High pH reversed-phase (RP) peptide fractionation: Peptides were dissolved in 100 μl of 9 mM ammonium formate, and 90 μl were loaded onto a XBridge Peptide BEH C18 column (Waters, 130 Å, 3.5 µm, 1 mm × 100 mm) on an Agilent 1200 HPLC. The samples were separated into 64 fractions using a gradient of 2% ACN, 9 mM ammonium formate (pH 10) to 40% ACN, 9 mM ammonium formate (pH 10) in 60 min at a flow rate of 1 ml/min and concatenated into 8 fractions before drying the peptides to completeness.

LC–MS Data Acquisition: LC–MS analysis of pooled high-pH RP fractions was conducted on a Q Exactive HF (Thermo Fisher Scientific) mass spectrometer operated in-line with an ACQUITY UPLC M-Class (Waters). A 75 µm forward-trap elute configuration was used for peptide separation on a 25 cm × 75 μm, 1.8 μm HSS T3 analytical column (Waters). The separating linear gradient covered 5% ACN, 0.1% FA to 35% ACN, 0.1% FA in 90 min at a flow rate of 400 nl/min. Mass spectra were essentially recorded in data dependent mode (top25). MS2 spectra were acquired by isolating peptide precursor ions at 0.7 Da, followed by HCD fragmentation at an NCE of 33 (AGC target: 1e5, maxIT: 87 ms, R: 45,000). Dynamic exclusion was set to 30 s and charge states of the type unassigned, 1, ≥ 6 was ignored.

Data analysis: Peptide and protein identification, as well as protein-level quantification relative to the lowest temperature (TMT channel 126), was conducted using Proteome Discoverer 2.5.0.4. Melting curve fitting was conducted using the Bioconductor TPP package 3.13.

### In vitro absorption, distribution, metabolism, and excretion – toxicity (ADMET) assays

In vitro ADME-T assays were performed by Cyprotex (Cyprotex Discovery, Cheshire, UK). *Semi-Thermodynamic solubility:* The shortlisted hits were added to a 96-well plate in quadruplicate and 1 × PBS was added to give a maximum concentration of 100 µM. The solution was agitated at ambient temperature overnight. The solutions were then centrifuged for 30 min at 3000 rpm at RT. The supernatant was removed and centrifuged for a further 30 min under the same conditions. An aliquot of the resulting supernatant was diluted in 50% methanol in water (containing an internal standard for MS analysis) prior to analysis by LC–MS/MS. A standard curve was produced by diluting the 10 mM DMSO stock with DMSO to give concentrations of 1 mM and 0.1 mM. These stocks were diluted in in 50% methanol in water (containing an internal standard for MS analysis) prior to analysis by LC–MS/MS. The solubility of the shortlisted hits was calculated from a linear or quadratic fit of the standard curve.

#### Microsomal metabolic stability

Pooled liver microsomes were purchased from Xenotech (H0500) and were stored according to manufacturer’s instructions prior to use. Microsomes (final protein concentration 0.5 mg/ml), 0.1 M phosphate buffer pH 7.4 and shortlisted hits (final substrate concentration 1 µM; final DMSO concentration 0.25%) were pre-incubated at 37 °C prior to addition of NADPH (final concentration 1 mM) to initiate the reaction. A minus co-factor control incubation was included for each compound tested where 0.1 M phosphate buffer pH 7.4 was added instead of NADPH. Two control compounds were included with each species. All incubations were performed singularly for each shortlisted hit. Each compound was incubated for 0, 5, 15, 30 and 45 min. The minus co-factor was incubated for 45 min only. The reactions were stopped by transferring incubate into acetonitrile at the appropriate time points in a 1:3 ratio. The termination plates were centrifuged at 3000 rpm for 20 min at 4 °C to precipitate the protein. Following protein precipitation, the supernatant were combined in cassette of up to 4 compounds, internal standard for MS analysis was added and samples were analyzed using LC–MS/MS. From a plot of In peak area ratio (compound peak area/internal standard peak area) against time, the gradient of the lines were determined. Subsequently, half-life and intrinsic clearance were calculated using the following equations.

$$\begin{array}{l}\mathrm E\mathrm l\mathrm i\mathrm m\mathrm i\mathrm n\mathrm a\mathrm t\mathrm i\mathrm o\mathrm n\;\mathrm r\mathrm a\mathrm t\mathrm e\;\mathrm c\mathrm o\mathrm n\mathrm s\mathrm t\mathrm a\mathrm n\mathrm t\;(\mathrm k)=(-\mathrm{gradient})\\\mathrm{Half}-\mathrm{life}(\mathrm t1/2)\;(\min)=0.693/\mathrm k\\\mathrm I\mathrm n\mathrm t\mathrm r\mathrm i\mathrm n\mathrm s\mathrm i\mathrm c\;\mathrm c\mathrm l\mathrm e\mathrm a\mathrm r\mathrm a\mathrm n\mathrm c\mathrm e\;(\mathrm{CLint})\;(\mathrm\mu\mathrm l/\min/\mathrm m\mathrm g\;\mathrm p\mathrm r\mathrm o\mathrm t\mathrm e\mathrm i\mathrm n)=\mathrm{Vx}0.693/{\mathrm t}_{1/2}\end{array}$$

where V = Incubation volume (µl) / Microsomal protein (mg)

#### CaCo-2 permeability (Bi-directional)

Caco-2 cells obtained from the ATCC were used between passage numbers 40—60. Cells were seeded onto Millipore Multiscreen Transwell plates at 1 × 10^5^ cells/cm^2^. The cells were cultured in DMEM and media was changed every two or three days. On day 20, the permeability study was performed. Cell culture and assay incubations were carried out at 37ºC in an atmosphere of 5% CO_2_ with a relative humidity of 95%. On the day of the assay, the monolayers were prepared by rinsing both apical and basolateral surfaces twice with Hanks Balanced Salt Solution (HBSS) at the desired pH warmed to 37 °C. Cells were then incubated with HBSS at the desired pH in both apical and basolateral compartments for 40 min to stabilize physiological parameters.

The dosing solutions were prepared by diluting the test compound with assay buffer to give a final compound concentration of 10 μM (final DMSO concentration of 1% v/v). The fluorescent integrity marker lucifer yellow was also included in the dosing solution. Analytical standards were prepared from test compound DMSO dilutions and transferred to buffer, maintaining a 1% v/v DMSO concentration.

For assessment of A-B permeability, HBSS was removed from the apical compartment and replaced with test compound dosing solution. The apical compartment insert was then placed into a companion plate containing fresh buffer (containing 1% v/v DMSO). For assessment of B-A permeability, HBSS was removed from the companion plate and replaced with test compound dosing solution. Fresh buffer (containing 1% v/v DMSO) was added to the apical compartment insert, which was then placed into the companion plate.

At 120 min, the apical compartment inserts and the companion plates were separated, and apical and basolateral samples were diluted for analysis.

Compound permeability was assessed in duplicate. Compounds of known permeability characteristics were run as controls on each assay plate.

Test and control compounds were quantified by LC–MS/MS cassette analysis using a 7-point calibration with appropriate dilution of the samples. The starting concentration (C_0_) was determined from the dosing solution and the experimental recovery calculated from C_0_ and both apical and basolateral compartment concentrations. The integrity of the monolayer throughout the experiment was checked by monitoring lucifer yellow permeation using fluorimetric analysis.

The permeability coefficient (Papp) for each compound was calculated from the following equation:

$${\mathrm{P}}_{\mathrm{app}}=\mathrm{dQ}/\mathrm{dtC}0\times \mathrm{A}$$

where dQ/dt was the rate of permeation of the drug across the cells, C_0_ was the donor compartment concentration at time zero and A was the area of the cell monolayer. C_0_ was obtained from analysis of the dosing solution.

Efflux ratio (ER) was calculated from mean A-B and B-A data. This was derived from:

$$\mathrm{ER}=\mathrm{Papp}(\mathrm{B}-\mathrm{A})\mathrm{Papp}(\mathrm{A}-\mathrm{B})$$

Three control compounds were screened alongside the test compounds, atenolol (human absorption 50%), propranolol (human absorption 90%) and talinolol (a substrate for P-glycoprotein).

#### Cell Viability (Cytotoxicity testing using HepG2)

HepG2 human hepatocellular carcinoma cells were plated on 96-well tissue culture polystyrene plates for 24 h prior to dosing of the cells. The shortlisted hits were diluted in DMSO and serial dilutions are made 1% DMSO in growth media. Compounds at 8 concentrations in triplicate was then incubated for 72 h. Appropriate blanks and controls were run alongside the assay. One h prior to the end of the incubation period, the cells were loaded with MTT [yellow; 3-(4,5-dimethyl-2-thiazolyl)-2,5-diphenyl-2H-tetrazolium bromide], the plates were dried and re-solubilized using DMSO. The plates were then scanned at 570 nm using a plate reader.

The minimum effective concentration was determined from the lowest concentration whose mean value exceeds the significance level, provided either a clear dose–response relationship was observed, or at least two consecutive concentration points were above the significance level.

### In vivo bioavailability analysis with E12, 3.14 and 7

In vivo bioavailability studies were performed by Pharmacology Discovery Services Taiwan, Ltd., (New Taipei City, Taiwan). Male ICR mice weighing 20—30 g were provided by BioLasco Taiwan (under Charles River Laboratories Licensee). Animals were acclimated for 3 days prior to use and were confirmed with good health. All animals were maintained in a hygienic environment with controlled temperature (20—24ºC), humidity (30%—70%) and 12 h light/dark cycles. Free access to sterilized standard lab diet [MFG (Oriental Yeast Co., Ltd., Japan)] and autoclaved tap water. A pharmacokinetic (PK) study was performed in male ICR mice following intravenous (IV) and oral (PO) administration of compound **7** and **E12** and IV administration of the test compound **3.14**. The three test compounds (**7**, **E12** and **3.14**) were formulated in 1% dimethyl sulfoxide (DMSO)/10% Solutol® HS-15/ phosphate buffered saline (PBS) at 0.2 mg/mL and 1 mg/mL for IV and PO administration, respectively. The dosing volumes were 5 ml/kg for IV and 10 ml/kg for PO. The dose was 1 mg/kg for IV and 10 mg/kg for PO routes. The plasma samples were collected and at 3, 10, 30, 60, 120, 240, 480, and 1440 min after IV and 10, 30, 60, 120, 240, 360, 480, and 1440 min after PO administration. t_1/2_ was calculated as follows:

$${t}_\frac{1}{2}=\frac{{ln}_{2}\bullet {V}_{D}}{CL}$$

### In vivo single dose and multi dose MTD studies with compound 7

In vivo MTD studies were performed by Pharmacology Discovery Services Taiwan (New Taipei City, Taiwan) Phase 1: Single-dose maximum tolerated dose (MTD): **7** was administered PO to groups of 2 male and 2 female (23 ± 3 g) ICR mice. Animals received an initial dose of 30 mg/kg. If the animals had no significant adverse effects within 60 min after treatment, the dose for the next cohort was increased. If one or more animals died or had significant adverse effects within 60 min after treatment, the dose for the next cohort was decreased. The testing stopped when all animals survived at the upper bound (200 mg/kg), or lower bound (3 mg/kg) had been reached. Full clinical examinations and body weight change were assessed. At each dose level, animals were observed, and mortality was noted daily after compound administration for three days. Animals were observed for the presence of acute toxic symptoms (mortality, convulsions, tremors, muscle relaxation, and sedation) and autonomic effects (diarrhea, salivation, lacrimation, vasodilation, piloerection, etc.) during the first 60 min and again at 120 min after administration. Body weights were recorded pre-dose and at 72 h.

The next dose level was determined by the following scheme:

- 30 mg/kg, if no death, 100 mg/kg, if no death, 200 mg/kg (upper bound)
- 30 mg/kg, if no death, 100 mg/kg, if death, 50 mg/kg
- 30 mg/kg, if death, 10 mg/kg, if death, 3 mg/kg (lower bound)
- 30 mg/kg, if death, 10 mg/kg, if no death, 17 mg/kg

Phase 2: **7** was administered PO at 200 mg/kg, bid × 5, 1 h interval, 200 mg/kg, qd × 5, and 150 mg/kg, bid × 5, 8-h interval to groups of 3 female CB.17 SCID mice (7 ± 1 week-old) for assessment of possible adverse effects. Animals were observed for the presence of acute toxic symptoms (mortality, convulsions, tremors, muscle relaxation, sedation) and autonomic effects (diarrhea, salivation, lacrimation, vasodilation, piloerection) during the first 50 (after 1^st^ daily dose) or 60 (2^nd^ daily dose) min after each dose. Body weights were recorded once daily for 8 days. The animals were observed for mortality twice daily for 8 days. In addition, plasma samples were collected at 0.5, 1, 2 and 5.3 h(s) after the second dose on Day 4 (150 mg/kg, PO; bid × 5 group) and at 0.5, 1, 2 and 6 h(s) after the final dose on Day 5 (200 mg/kg, PO; qd × 5 group). The exposure levels (ng/mL) of **7** in plasma samples were then determined by LC–MS/MS. *AUClast* was calculated from the area under the plasma concentration–time curve from time zero to time of last measurable concentration.

### In vivo PK studies with 7

In vivo PK studies were performed by Pharmacology Discovery Services Taiwan (New Taipei City, Taiwan). **7** (30, 100, and 200 mg/kg, PO) was further administered to groups of 2 male and 2 female (23 ± 3 g) satellite ICR mice in PK study; the plasma and liver samples were harvested at 0.5 h after administration. In addition, one additional group was dosed at 30 mg/kg and the plasma samples were harvested at 1 and 2 h(s) after the treatment. The body and liver weights were recorded. The exposure levels (ng/ml or ng/g) of **7** in plasma and liver samples were then determined by Liquid Chromatograph Tandem Mass Spectrometer (LC–MS/MS), and the plasma: liver ratios were calculated.

### Anti-tumor activity in mouse models

In vivo mouse models were generated and experiment with SKOV3 and AGS models performed by EPO, experimental pharmacology & oncology Berlin-Buch GMBH (Berlin, Germany). AGS model: AGS cells were cultured in F12-K medium (10%FCS, 1%PS) until P11. Freshly isolated cells (1 × 10^6^) were subcutaneously transplanted to left flank of 8-week-old female mice in PBS/Matrigel (1:1). After achieving a TV group mean of 0.139 and 0.140cm3, mice were randomly assigned to three study groups with n = 6 animals (TV: min. 0.106 / max. 0.229cm^3^) and were daily treated with either the vehicle (1% DMSO/ 10% solutol/ PBS), **7** (200 mg/kg in 1% DMSO / 10% solutol / PBS) or BGJ398 (10 mg/kg, in 2:1 PEG300/D5W). Tumor diameters were determined by calliper measurements 2 × weekly, body weight (BW) was measured as parameter for tolerability 2 × weekly.

SKOV3 model: SKOV3 cells were cultured in McCoy´s 5A (26,600–023, ThermoFisher) media (10%FCS) until P7. Freshly isolated cells (1 × 10^6^) with a viability of 98% were subcutaneously transplanted to left flank of 8-week-old female mice in PBS/Matrigel (1:1). After achieving a TV group mean of 0.138 and 0.143cm^3^ mice were randomly assigned to three study groups with n = 8 animals (TV: min. 0.102 / max. 0.216cm^3^) after 48 h post cell transplantation and were daily treated either the vehicle (1% DMSO/ 10% solutol/ PBS), **7** (200 mg/kg in 1% DMSO / 10% solutol / PBS) or BGJ398 (10 mg/kg, in 2:1 PEG300/D5W). Tumor diameters were determined by calliper measurements 2 × weekly, body weight (BW) was measured as parameter for tolerability 2 × weekly. For assessing significance of differences between treatments at the individual timepoints, a two-way ANOVA analysis with Turkey’s multiple comparison test was performed.

### Immunohistochemistry (IHC)

IHC of tumor sections was performed by Sophistolab (Muttenz, Switzerland) on a Lecia BondMax instrument using Refine HRP-Kits (Leica DS9800). All buffer-solutions were purchased from Lecia Microsystems Newcastle, Ltd and used according to the manufacturer’s guidelines. Paraffin-slides were de-waxed, pre-treated and incubated as follows: ER-solution 2 for 10 min at 95 °C, ER-solution 2 for 20 min at 100 °C and ER-solution 2 for 30 min at 100 °C. pFRS2; Rabbit anti-Phospho FRS2 (PhosphoY 436, Abcam Limited; ab193363), dilution 1:150; phospho-p44/42 MAPK (Erk1/2_Thr202/Tyr204_) Rabbit mAb (Cell Signaling Technology #4370), dilution 1:1600. The IHC images were captured digitally using a Nikon Epifluorescence Eclipse Ti2 equipped with a Nikon DS-Ri2 color and a Nikon DS-Qi2 monochrome camera.

### Quantification and statistical analysis

Mean ± SEM are shown when means of three biological replicas are compared, mean and SD when three technical replicas are compared. Unpaired student’s t-test was used to test significance of differences between two samples. For all other analyses, one-way ANOVA repeated measures test using Bonferroni's Multiple Comparison with Prism software was performed. *P* values or adjusted *P* values < 0.05 were considered significant (ns *p* > 0.05, * *p* ≤ 0.05, ** *p* ≤ 0.01, *** *p* ≤ 0.001, **** *p* ≤ 0.0001). Where indicated, asterisks show statistical significances between control and test sample.

## Supplementary Information

Below is the link to the electronic supplementary material.

Supplementary file1 (DOCX 16845 KB)

Supplementary file2 (DOCX 775 KB)

Supplementary file3 (XLSX 3673 KB)

Supplementary file4 (XLSX 25 KB)

Supplementary file5 (XLSX 15 KB)

Supplementary file6 (XLSX 8843 KB)

Supplementary file7 (XLSX 4368 KB)

## Data Availability

All data generated or analysed during this study are included in this published article [and its supplementary information files]. The raw datasets used and/or analysed during the current study are available from the corresponding author on reasonable request.

## Abbreviations

aCDc: Automated cell dissemination counter
ADMET: Absorption, distribution, metabolism, and excretion – toxicity
ALK: Anaplastic Lymphoma Kinase
AKT: AKr mouse strain Thymoma
AUClast: Area under the plasma concentration–time curve from time zero to time of last measurable concentration
BW: Body weight
CETSA: Cellular thermal shift assay
CSP: Chemical shift perturbation
CTNNA3: Catenin alpha 3
DMSO: Dimethylsulfoxid
DSF: Differential scanning fluorimetry
EGFR: Epidermal growth factor receptor
FGFR: Fibroblast growth factor receptor
FRS2: Fibroblast growth factor receptor substrate 2
GB1: Streptococcal protein G1
HBSS: Hanks Balanced Salt Solution
HGF: Hepatocyte growth factor
IB: Immunoblotting
IHC: Immunohistochemistry
IPTG: Isopropyl-β-D-thiogalactopyranosid
IV: Intravenous
JAK: Janus kinase
LC–MS/MS: Liquid chromatography with tandem mass-spectrometry
MAPK: Mitogen-activated protein kinase
MET: Mesenchymal epithelial transition
MB: Medulloblastoma
MST: Microscale thermophoresis
MTD: Maximum tolerated dose
MTT: 3-(4, 5-Dimethylthiazol-2-yl)-2, 5-diphenyltetrazolium bromide
NMR: Nuclear magnetic resonance
PBS: Phosphate buffered saline
PI3-K: Phosphatidylinositol 3’-kinase
PLC-g: Phospho-lipase gamma
PK: Pharmacokinetic
PO: Peroral
PTB: Protein tyrosine binding
PPI: Protein-protein interaction
RAS: Rat sarcoma virus
RP: Reversed-phase
RTK: Receptor tyrosine kinase
SHH: Sonic hedgehog
SIA: Spheroid invasion assay
STD: Saturation transfer difference
TAAC: Transforming acidic coiled-coil containing protein 3
TPP: Thermal proteome profiling
TV: Tumor volume
WaterLOGSY: Water-ligand observed via gradient spectroscopy
