# Supplementary material for: Discovery of a small molecule ligand of FRS2 that inhibits invasion and tumor growth
Source: Cell Oncol (Dordr). 2022 Dec 10;46(2):331–56. doi: 10.1007/s13402-022-00753-x (PMC10060354; doi:10.1007/s13402-022-00753-x)
Supplement: Supplementary file 1 — Supplementary file1 (DOCX 16845 KB) [file 13402_2022_753_MOESM1_ESM.docx]

**Discovery of a small molecule ligand of FRS2 that inhibits invasion and tumor growth**

**Authors**

Karthiga Santhana Kumar^1,6^, Cyrill Brunner^2^, Matthias Schuster^3^, Levi Luca Kopp^1^, Alexandre Gries^1^, Shen Yan^1^, Simon Jurt^3^, Kerstin Moehle^3^, Dominique Bruns^2^, Michael Grotzer^4^, Oliver Zerbe^3^, Gisbert Schneider^2,5^ and Martin Baumgartner^1*^

**Supplementary figures**

**FIG. S1**

**a**) Structures of compounds tested in SIA in figure 1a. **b)** Structural modifications in 3.18 analogs.

**FIG. S2**

**a)** Structures of E-series compounds. **b)** Quantification of representative spheroid invasion assay (SIA) in DAOY cells treated with indicated E-series compounds at 10 µM concentration. Violin plot with median and quartiles of distances of invasion and adjusted p-values from representative experiment are shown. Red dotted line: Maximal repression of bFGF-induced invasion, green dotted line: Maximal bFGF-induced invasion. **c)** Schematic representation of the structures of FRS2-PTB proteins expressed in *E.coli* and used in this study. **d**) Plotted first derivatives of nanoDSF analysis of GB1-FRS2_PTB or GB1 alone or in combination with FGFR_PEP across temperature gradient recorded at 350 nm. **e) EC50** of **E12** and **E25** for collagen I invasion inhibition in DAOY cells. Log(Y) transformed and normalized invasion distances and corresponding SD of n = 3 technical replicas are plotted against compound concentrations.

**
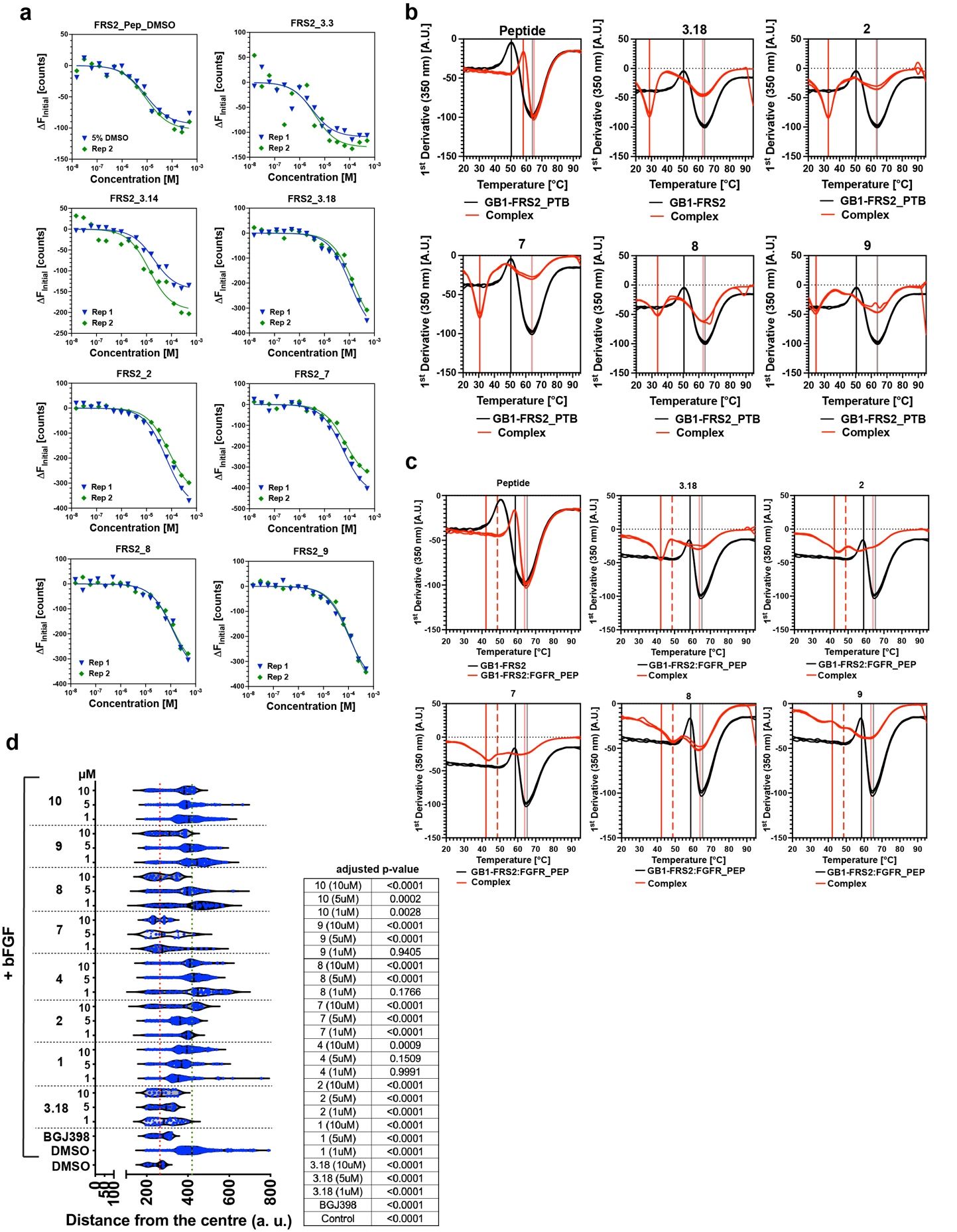
**

**FIG. S3**

**a)** MST analysis by initial fluorescence change of compounds **3.3**, **3.14** and **3.18** as well as analogs of **3.18**. **b**) Plotted first derivatives of nanoDSF analysis of GB1-FRS2_PTB or GB1 in the presence of **3.18** and structural analogs of **3.18** recorded at 350 nm. **c**) Competition assays of compounds against FGFR_PEP. Plotted first derivatives of nanoDSF analysis recorded at 350 nm of GB1-FRS2_PTB are shown. Black: GB1-FRS2_PTB:FGFR_PEP, Red: GB1-FRS2_PTB:FGFR_PEP plus added compound. **d**) Quantification of SIA in DAOY cells of structural **3.18** analogs. Violin plot with median and quartiles of distances of invasion and adjusted p-values from representative experiment with n = 3 technical replicas are shown.

**FIG. S4**

**a)** Quantification of SIA analysis with bFGF-stimulated DAOY cells comparing anti-invasion efficacies of shortlisted compounds at 10 µM concentration. SIA analysis with HGF- **(b)** or EGF-stimulated **(c)** DAOY cells comparing anti-invasion efficacies of shortlisted compounds at 10 µM concentration. **d)** SIA analysis with unstimulated ONS-76 cells comparing anti-invasion efficacies of shortlisted compounds at 10 µM concentration. **e)** Heat map indicating relative change in invasion expressed as % of unstimulated control. Mean and SD of invasion distances from n = 3 biological replicas are shown in a – c. *** = p<0.001, **** = p<0.0001 of one-way ANOVA with Dunnett’s multiple comparison test.

**FIG. S5**

Screening data for compounds **3.14** **a)** and **13** **b**). Reference, STD and WATERLOGSY spectra are depicted. **c**) [^15^N,^1^H]-HSQC spectra of various FRS2 constructs. All spectra were recorded at 600 MHz, 25 ºC. I: FRS2_PTB. II: FRS2_PTB + 2 equiv. FGRF1_PEP in 20 mM CHAPS. III: GB1-FRS2_PTB. IV: GB1-FRS2_PTB-FGRF1-Pep. **d**) Assignment status. Residues for which backbone assignments were made are color-coded in red (protein) or yellow (FGFR1) on the structure of the non-covalent complex (pdb entry 1XR0). Assigned protein and peptide linker residues are depicted in red and yellow, respectively. Residues experiencing larger CSPs are depicted by sticks. **e**) Comparison of the CSPs observed upon interaction with compounds **3.14**, **7** and **13**. FRS2-PTB residues are in grey, FGFR1_PEP in yellow. Residues experiencing larger CSPs are depicted by sticks in cyan.


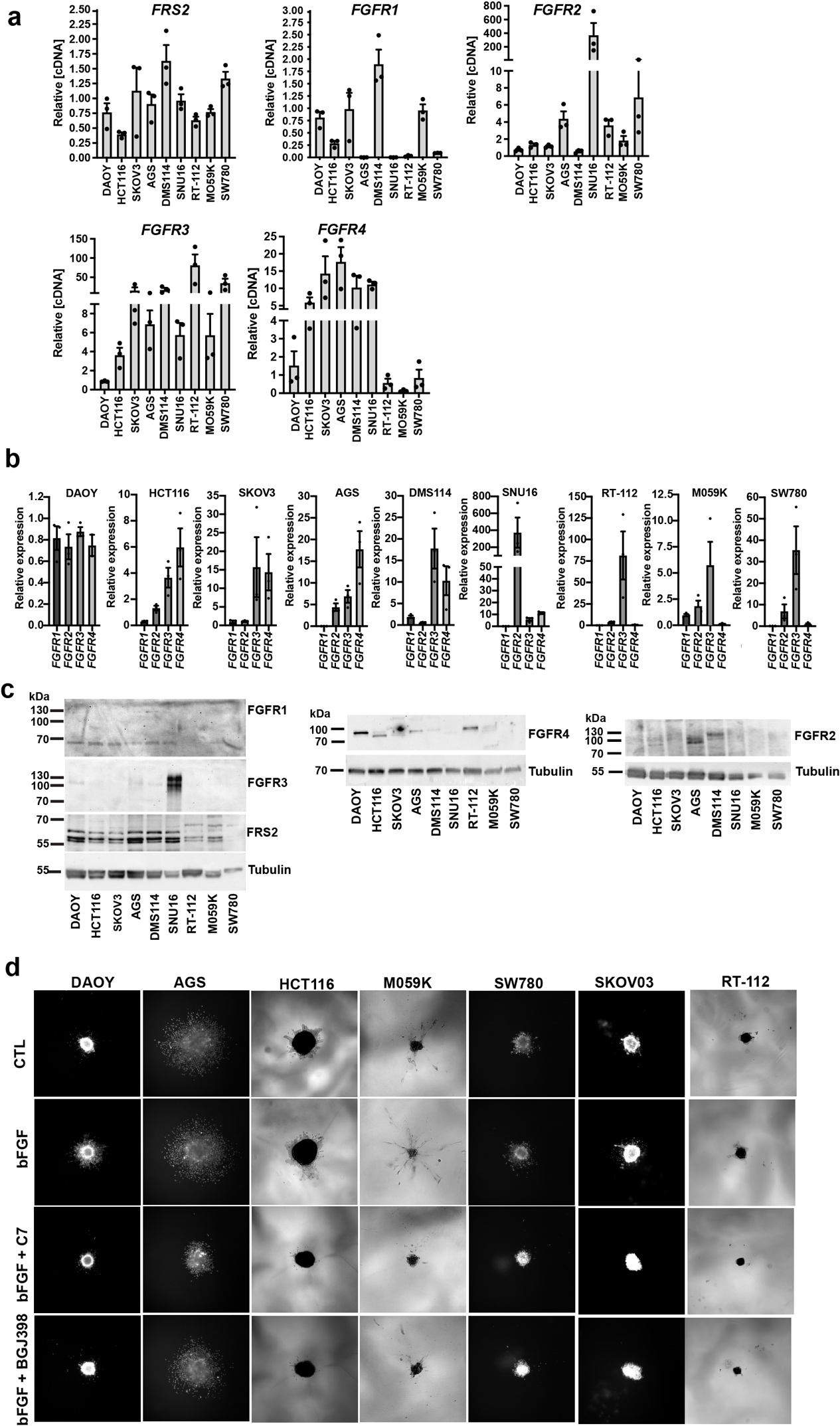


**FIG. S6**

**a)** RT-qPCR analysis of *FRS2* and *FGFR* expression across cancer cell lines used. Mean and SD of n = 3 biological replicas is shown. **b)** Comparative rt-qPCR analysis of *FGFR* expression by cell line. Mean and SD of n = 3 biological replicas are shown. **c)** IB analysis of FRS2 and FGFR expression across cell lines. **d)** Representative images of SIA of cell lines with indicated treatments at assay endpoint.

**Figure S7**

**a)** Heat maps of cellTiter-Glo assays of cancer cell lines grown in 2D cultures depicting viability as % luminescence of control. **b)** Heat maps of 3D cellTiter-Glo assays of cancer cell lines grown in 3D cultures depicting viability as % luminescence of control. **c)** IB analysis of ERK1/2 (Thr202/Tyr204) phosphorylation in compound treated SKOV3 cells stimulated with bFGF. **d)** IB analysis of ERK1/2 (Thr202/Tyr204) phosphorylation in compound treated AGS cells stimulated with bFGF. **e)** IB analysis of ERK1/2 (Thr202/Tyr204) phosphorylation in compound treated HCT116 cells stimulated with bFGF. **f,g)** IB analysis of ERK1/2 (Thr202/Tyr204) and AKT (S476) phosphorylation in compound treated DMS114 (f) and RT-112 (g) cells stimulated with bFGF. **g)** Bar diagrams in c-f depict quantifications of ERK1/2 (Thr202/Tyr204) phosphorylation relative to unstimulated control. Mean fold change of phosphorylation of n = 3 independent experiments, SD and one-way ANOVA adjusted p-values of comparison to DMSO+bFGF are shown. * = p<0.0%, ** = p<0.01, *** = p<0.001, **** = p<0.0001.

**Figure S8**

**a**) IC_50_ curves of viability of cancer cell lines determined by CellTiterGlo assay after treatment with increasing concentrations of compound 7 in 2D cell viability assay. Means and SD of n = 3 technical replicas are shown. **b**) Exposure of compound 7 in plasma and liver 0.5 h after PO gavage of 7. Means and SD of n = 4 animals are shown. **c**) Plasma exposure levels during 5x q.d. PO gavage of 200 mg/kg compound 7 at indicated times after gavage. Means and SD of n = 3 animals are shown. **d**) Timeline and treatment scheme in SK-OV-3 mouse flank model. **e)** Averaged growth curves with mean slopes of SKOV3 tumors shown in Fig. 5d. **f**) Tumor volume (TV) and body weight (BW) measurements in 6 mice per condition in SKOV3 model. **g**) Timeline and treatment scheme in AGS mouse flank model. **h**) Tumor volume (TV) and body weight (BW) measurements in 6 mice per condition in AGS model.

**Figure S9**

**a**) Nonlinear fit of FRS2 and tubulin abundance of CETSA from DMSO or compound 7-treated DAOY cells. **b**) Upper: Scatterplot of all proteins with high quality melting curves in whole cell TPP analysis from both conditions. Lower: Bar plot with gene symbols of proteins with altered *T_m_* (p<0.05). Red bars ∆*T_m_* ≥ -5°C, green bars: ∆*T_m_* ≥ +5°C. **c**) Upper: Scatterplot of all proteins with high quality melting curves in lysate TPP analysis from both conditions. Lower: Bar plot with gene symbols of proteins with altered *T_m_* (p<0.05). Red bars ∆*T_m_* ≥ -5°C, green bars: ∆*T_m_* ≥ +5°C.
