# Supplementary material for: Discovery of a small molecule ligand of FRS2 that inhibits invasion and tumor growth
Source: Cell Oncol (Dordr). 2022 Dec 10;46(2):331–56. doi: 10.1007/s13402-022-00753-x (PMC10060354; doi:10.1007/s13402-022-00753-x)
Supplement: Supplementary file 2 — Supplementary file2 (DOCX 775 KB) [file 13402_2022_753_MOESM2_ESM.docx]

**Discovery of a small molecule ligand of FRS2 that inhibits invasion and tumor growth**

**Authors**

Karthiga Santhana Kumar^1,6^, Cyrill Brunner^2#^, Matthias Schuster^3#^, Levi Luca Kopp^1#^, Alexandre Gries^1^, Shen Yan^1^, Simon Jurt^3^, Kerstin Moehle^3^, Dominique Bruns^2^, Michael Grotzer^4^, Oliver Zerbe^3^, Gisbert Schneider^2,5^ and Martin Baumgartner^1*^

**Supplementary Materials**

6His-GB1-FRS2_PTB_FGFR1_pep (NMR assignments):

atgtctggttctcatcatcatcatcatcatagcagcggcatcgaaggccgcggccgccagtacaaactgatcctgaacggtaaaaccctgaaaggtgaaaccaccaccgaagctgttgacgctgctaccgcggaaaaagttttcaaacagtacgctaacgacaacggtgttgacggtgaatggacctacgacgacgctaccaaaaccttcaccgttaccgaaagcagcggcgaaaacctgtacttccagggagataccgtgcctgacaatcatcgcaataagtttaaagtaatcaacgtcgatgatgatggcaacgagttgggtagcgggatcatggagctgacggacacagaacttatcctgtatactcgtaaacgtgattctgttaaatggcactacttgtgtttgcgtcgctatggatacgactcgaatttattctcatttgaaagtggtcgtcgctgccagaccgggcagggaatttttgcttttaagtgtgcgcgtgcggaagagttgttcaatatgttacaggaaatcatgcaaaacaattcgatcaacgtcgttgaggaacccgtcgtcgaataataaggatccggctgctaacaaagctcggggtcaagtggaagctcaggctcgtcgggcagctcagggcatagtcagatggcggtacacaaattggcgaaatcaatccctctgcgccgccaggtcaccgtatcc

atgtctggttctcatcatcatcatcatcatagcagcggcatcgaaggccgcggccgccag
 M  S  G  S  H  H  H  H  H  H  S  S  G  I  E  G  R  G  R  Q 
tacaaactgatcctgaacggtaaaaccctgaaaggtgaaaccaccaccgaagctgttgac
 Y  K  L  I  L  N  G  K  T  L  K  G  E  T  T  T  E  A  V  D 
gctgctaccgcggaaaaagttttcaaacagtacgctaacgacaacggtgttgacggtgaa
 A  A  T  A  E  K  V  F  K  Q  Y  A  N  D  N  G  V  D  G  E 
tggacctacgacgacgctaccaaaaccttcaccgttaccgaaagcagcggcgaaaacctg
 W  T  Y  D  D  A  T  K  T  F  T  V  T  E  S  S  G  E  N  L 
tacttccagggagataccgtgcctgacaatcatcgcaataagtttaaagtaatcaacgtc
 Y  F  Q  G  D  T  V  P  D  N  H  R  N  K  F  K  V  I  N  V 
gatgatgatggcaacgagttgggtagcgggatcatggagctgacggacacagaacttatc
 D  D  D  G  N  E  L  G  S  G  I  M  E  L  T  D  T  E  L  I 
ctgtatactcgtaaacgtgattctgttaaatggcactacttgtgtttgcgtcgctatgga
 L  Y  T  R  K  R  D  S  V  K  W  H  Y  L  C  L  R  R  Y  G 
tacgactcgaatttattctcatttgaaagtggtcgtcgctgccagaccgggcagggaatt
 Y  D  S  N  L  F  S  F  E  S  G  R  R  C  Q  T  G  Q  G  I 
tttgcttttaagtgtgcgcgtgcggaagagttgttcaatatgttacaggaaatcatgcaa
 F  A  F  K  C  A  R  A  E  E  L  F  N  M  L  Q  E  I  M  Q 
aacaattcgatcaacgtcgttgaggaacccgtcgtcgaatcggggtcaagtggaagctca
 N  N  S  I  N  V  V  E  E  P  V  V  E  S  G  S  S  G  S  S

ggctcgtcgggcagctcagggcatagtcagatggcggtacacaaattggcgaaatcaatc

 G  S  S  G  S  S  G  H  S  Q  M  A  V  H  K  L  A  K  S  I

Cctctgcgccgccaggtcaccgtatcc

 P  L  R  R  Q  V  T  V  S

6xHis

GB1

TEV

GS linker

FRS2-PTB 11-140aa

hFGFR1 409-430

6His-GB1-FRS2_PTB (nanoDSF, MST, NMR):

Atgtctggttctcatcatcatcatcatcatagcagcggcatcgaaggccgcggccgccagtacaaactgatcctgaacggtaaaaccctgaaaggtgaaaccaccaccgaagctgttgacgctgctaccgcggaaaaagttttcaaacagtacgctaacgacaacggtgttgacggtgaatggacctacgacgacgctaccaaaaccttcaccgttaccgaaagcagcggcgaaaacctgtacttccagggagataccgtgcctgacaatcatcgcaataagtttaaagtaatcaacgtcgatgatgatggcaacgagttgggtagcgggatcatggagctgacggacacagaacttatcctgtatactcgtaaacgtgattctgttaaatggcactacttgtgtttgcgtcgctatggatacgactcgaatttattctcatttgaaagtggtcgtcgctgccagaccgggcagggaatttttgcttttaagtgtgcgcgtgcggaagagttgttcaatatgttacaggaaatcatgcaaaacaattcgatcaacgtcgttgaggaacccgtcgtcgaataataaggatccggctgctaacaaagc

atgtctggttctcatcatcatcatcatcatagcagcggcatcgaaggccgcggccgccag
 M  S  G  S  H  H  H  H  H  H  S  S  G  I  E  G  R  G  R  Q 
tacaaactgatcctgaacggtaaaaccctgaaaggtgaaaccaccaccgaagctgttgac
 Y  K  L  I  L  N  G  K  T  L  K  G  E  T  T  T  E  A  V  D 
gctgctaccgcggaaaaagttttcaaacagtacgctaacgacaacggtgttgacggtgaa
 A  A  T  A  E  K  V  F  K  Q  Y  A  N  D  N  G  V  D  G  E 
tggacctacgacgacgctaccaaaaccttcaccgttaccgaaagcagcggcgaaaacctg
 W  T  Y  D  D  A  T  K  T  F  T  V  T  E  S  S  G  E  N  L 
tacttccagggagataccgtgcctgacaatcatcgcaataagtttaaagtaatcaacgtc
 Y  F  Q  G  D  T  V  P  D  N  H  R  N  K  F  K  V  I  N  V 
gatgatgatggcaacgagttgggtagcgggatcatggagctgacggacacagaacttatc
 D  D  D  G  N  E  L  G  S  G  I  M  E  L  T  D  T  E  L  I 
ctgtatactcgtaaacgtgattctgttaaatggcactacttgtgtttgcgtcgctatgga
 L  Y  T  R  K  R  D  S  V  K  W  H  Y  L  C  L  R  R  Y  G 
tacgactcgaatttattctcatttgaaagtggtcgtcgctgccagaccgggcagggaatt
 Y  D  S  N  L  F  S  F  E  S  G  R  R  C  Q  T  G  Q  G  I 
tttgcttttaagtgtgcgcgtgcggaagagttgttcaatatgttacaggaaatcatgcaa
 F  A  F  K  C  A  R  A  E  E  L  F  N  M  L  Q  E  I  M  Q 
aacaattcgatcaacgtcgttgaggaacccgtcgtcgaa
 N  N  S  I  N  V  V  E  E  P  V  V  E

6xHis

GB1

TEV

GS linker

FRS2-PTB 11-140aa

FRS2-FLAG:


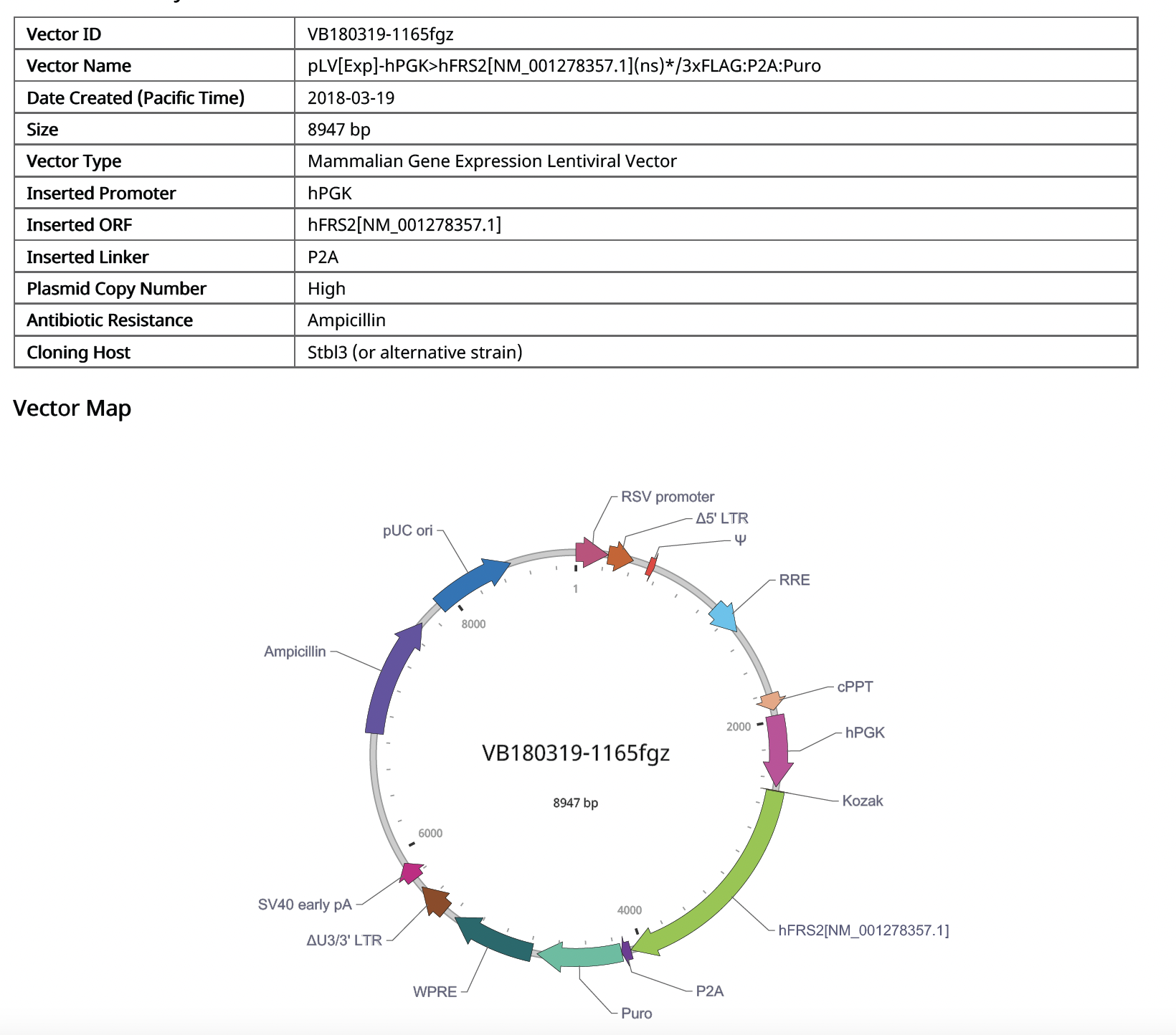


**KEY RESOURCES TABLE**

| REAGENT or RESOURCE | SOURCE | | IDENTIFIER |
| --- | --- | --- | --- |
| **Antibodies** | | | |
| Anti-FGFR1 | Cell signaling technologies | | 3472S |
| Anti-FGFR2 | Cell signaling technologies | | 11835S |
| Anti-FGFR4 | Santa Cruz | | SC-124 |
| Anti-GFAP | Abcam | | Ab53554 |
| Anti-AKT | Cell signaling technologies | | 927S |
| Anti-pAKT(S473) | Cell signaling technologies | | 4060L |
| Anti-phospho ERK1/2 (Thr202/Tyr204), | Cell signaling technologies | | 4370 |
| Anti-ERK1/2 | Cell signaling technologies | | 9102 |
| Anti-phospho FRS2 (Y436) (IB) | Cell signaling technologies | | 3861S |
| Anti-phospho FRS2 (Y436) (IHC) | Abcam | | ab193363 |
| Anti-FRS2 | Abcam | | Ab137458 |
| Anti-beta-tubulin | Sigma Aldrich | | T5201 |
| Anti-mouse horseradish peroxidase (HRP) linked | Cell signaling technologies | | 7076 |
| Anti-rabbit HRP linked | Cell signaling technologies | | 7074 |
| Anti-tubulin | Sigma Aldrich | | T9026 |
| Anti-human nuclei | Merck mmunere | | MAB4383 |
| Anti-rabbit-Cy3-coupled | Jackson mmune research | | 711-165-152 |
| Anti-mouse-Cy5-coupled | Jackson mmune research | | 415-175-166 |
|  |  | |  |
| **Bacterial and Virus Strains** |  | |  |
| LentiCRISPR | McComb S, *et al.* Sci Transl Med. 2016;8. | | N/A |
| pVSV | Oliver Pertz | | N/A |
| pMDL | Oliver Pertz | | N/A |
| pRev | Oliver Pertz | | N/A |
| pLA-EGFP | Oliver Pertz | | N/A |
| **Biological Samples** |  | |  |
| Paraffin embedded xenograft samples from SK-OV-3 and AGS mouse models | EPO berlin | | N/A |
| **Chemicals, Peptides, and Recombinant Proteins** |  | |  |
| basic Fibroblast Growth Factor | PeproTech | | 100-18B |
| Epidermal growth factor | PeproTech | | AF-100-15 |
| Hepatocyte growth factor | PeproTech | | 100-39H |
| BGJ398 | Selleckchem | | S2183 |
| Hoechst (, Sigma-Alrich) | Sigma Aldrich | | B2883 |
| Pure coll® bovine collagen 1 | Cell systems | | 5005-B |
| Glycergel | Dako | C0563 | |
| **Critical Commercial Assays** |  | |  |
| CellTiterGlo | Promega | | G9241 |
| RNeasy Mini RNA isolation kit | Qiagen | | 74106 |
| High capacity cDNA reverse transcription kit | Thermo Fisher | | 4368814 |
| **Deposited Data** |  | |  |
| Not applicable |  | |  |
| **Experimental Models: Cell Lines** |  | |  |
| DAOY, Desmoplastic MB, age of patient: 4 years, gender: male, authenticated using genotyping | ATCC | |  |
| ONS-76, SHH medulloblastoma | Michael Taylor lab | |  |
| RT112, urinary bladder carcinoma | Leibniz-Institut  DSMZ-Deutsche Sammlung von Mikroorganismen und Zellkulturen GmbH | | ACC418 |
| AGS, gastric adenocarcinoma | ATCC, LGC | | CRL-1739 |
| DMS 114, Small cell lung cancer | ATCC, LGC | | CRL-2066 |
| HCT 116, colorectal carcinoma | ATCC, LGC | | CCL-247 |
| KATO III, gastric carcinoma | ATCC, LGC | | HTB-103 |
| KG-1, acute myelogenous leukemia | ATCC, LGC | | CCL-246 |
| SNU-16, gastric carcinoma | ATCC, LGC | | CRL-5974 |
| SW780, urinary bladder carcinoma | ATCC, LGC | | CRL-2169 |
| SW-837, grade IV adenocarcinoma | ATCC, LGC | | CCL-235 |
| Mice for ex vivo experiments – C57BL/6JRj, pregnant female, male and female pubs were used for brain dissection. Sex of pubs was not determined and slices were randomised. | Janiver labs | | N/A |
| Mice for *in vivo* experiments – | Eurofins, EPO | |  |
| **Oligonucleotides** |  | |  |
| FRS2 | ThermoFisher Sci. | | Hs00183614_m1 |
| GAPDH | ThermoFisher Sci. | | Hs02758991_g1 |
| FGFR1 | ThermoFisher Sci. | | Hs00915142_m1 |
| FGFR2 | ThermoFisher Sci. | | Hs01552926_m1 |
| FGFR3 | ThermoFisher Sci | | Hs00179829_m1 |
| FGFR4 | ThermoFisher Sci | | Hs01106908_m1 |
| **siRNAs** |  | |  |
| siCTL1 (silencer select, neg. CTL1) | Invitrogen | | 4390843 |
| siFRS2_1 (Silencer Select FRS2) | Invitrogen | | s21261 |
| siCTL2 (Stealth RNAi siRNA neg.CTL2) | Invitrogen | | 12935300 |
| siFRS2_2 (Stealth RNAi siRNA FRS2) | Invitrogen | | HSS116706 |
| **Software and Algorithms** |  | |  |
| Automated cell dissemination counter (aCDc) platform. Automated spheroid dissemination counter software (asDICs) used for quantification. | Kumar et al., 2015a | | <http://www.infozentrum.ethz.ch/uploads/user_upload/Software/> |
| R2 microarray | Kumar K.S. et al., 2015b and this paper | | <http://r2.amc.nl> |
|  |  | |  |
|  |  | |  |
|  |  | |  |
